# Nucleosomal Histone Proteins of *L. donovani*: A Combination of Recombinant H2A, H2B, H3 and H4 Proteins Were Highly Immunogenic and Offered Optimum Prophylactic Efficacy against *Leishmania* Challenge in Hamsters

**DOI:** 10.1371/journal.pone.0097911

**Published:** 2014-06-13

**Authors:** Rajendra K. Baharia, Rati Tandon, Amogh A. Sahasrabuddhe, Shyam Sundar, Anuradha Dube

**Affiliations:** 1 Division of Parasitology, Central Drug Research Institute, Lucknow, India; 2 Division of Molecular and structural Biology, Central Drug Research Institute, Lucknow, India; 3 Department of Medicine, Institute of Medical Sciences, Banaras Hindu University, Varanasi, India; INRS - Institut Armand Frappier, Canada

## Abstract

The present study includes cloning and expression of recombinant *Leishmania donovani* histone proteins (rLdH2B, rLdH3, rLdH2A and rLdH4), assessment of their immunogenicity in *Leishmania* infected cured patients/endemic contacts as well as in cured hamsters and finally evaluation of their prophylactic efficacy in hamsters against *L. donovani* challenge. All recombinant proteins were expressed and purified from the heterologous bacterial host system. *Leishmania* infected cured patients/endemic contacts as well as cured hamsters exhibited significantly higher proliferative responses to individual recombinant histones and their pooled combination (rLdH2B+rLdH3+rLdH2A+rLdH4) than those of *L.donovani* infected hosts. The *L.donovani* soluble antigens (SLD) stimulated PBMCs of cured/exposed and *Leishmania* patients to produce a mixed Thl/Th2-type cytokine profile, whereas rLdH2B, rLdH3, rLdH2A, rLdH4 and pooled combination (rLdH2-4) stimulated the production of Th1 cytokines IFN-γ, IL-12 and TNF-α but not Th2 cytokines IL-4 or IL-10. The immunogenicity of these histone proteins along with their combination was also checked in cured hamsters where they stimulated higher lymphoproliferation and Nitric oxide production in lymphocytes of cured hamsters than that of infected controls. Moreover, significantly increased IgG2 response, an indicative of cell mediated immunity, was observed in cured hamsters against these individual proteins and their combination as compared to infected hamsters. Further, it was demonstrated that rLdH2B, rLdH3, rLdH2A and rLdH4 and pooled combination were able to provide considerable protection for hamsters against *L. donovani* challenge. The efficacy was supported by the increased inducible Nitric Oxide Synthase (iNOS) mRNA transcripts and Th1-type cytokines - IFN-γ, IL-12 and TNF-α and down-regulation of IL-4, IL-10 and TGF-β. Hence, it is inferred that pooled rLdH2-4 elicits Thl-type of immune responses exclusively and confer considerable protection against experimental Visceral Leishmaniasis.

## Introduction

Visceral leishmaniasis (VL) is one of the most severe forms of Leishmanases caused by an intracellular protozoan parasite of the *Leishmania donovani/L. infantum/L. chagasi* complex and transmitted through the bites of the sand fly *Phlebotomus argentipes*. The disease is known to cause progressive fatal disease in poor people particularly belonging to North-eastern part of Indian sub-continent. An average of more than 90% of VL cases in India is reported from Bihar alone [1], [2]. Recent epidemics of VL in Sudan and India have resulted in over 100,000 deaths [3]. VL is characterized by prolonged fever, hepatosplenomegaly, anaemia, weightloss, cachexia, pancytopenia, hypergammaglobulinemia and suppressed cellular immune response [4]. Due to the non-availability of an ideal antileishmanial drug 350 million people are globally at the risk of acquiring infection with Leishmania parasites worldwide [5]. The disease is also emerging as an important opportunistic infection in immunocompromised patients, especially those co-infected with HIV [6], [7], [8].

The situation has further worsened with the emergence of drug resistance in various regions of endemicity and improper chemotherapeutic treatment of visceral leishmaniasis [6]. Current VL therapy is based on the long-term parenteral administration of pentavalent antimonials, which, despite being expensive and highly toxic, has been the standard treatment for over 50years. Currently available drugs are not safe for long term continual use for being highly cost ineffective. Due to the lack of effective and low-cost treatments, it is imperative to develop an ideal vaccine which would be protective against VL. So far killed or live-attenuated parasites, as well as a large number of *Leishmania* antigens from different species either in DNA or protein form have been tested as vaccines against VL with little success.

In current scenario, there has been significant progress in understanding the immuno-pathogenesis of leishmaniasis. The parasite antigen able to induce an immune response has been predominantly associated with the identification of proteins that may be used for vaccine development. Most of the studies aimed at identifying antigens from *Leishmania spp* have searched for molecules with the ability to stimulate Th1-type responses, which are known to be the major defense mechanism against *Leishmania* infection [9], [10], [11], [12], [13], [14], [15], [16].

Histone proteins- H2A, H2B, H3 & H4 which play an important role in DNA packaging, transcription and gene regulation, have been reported previously as potent vaccine candidate against cutaneous and viscerocutaneous leishmaniasis [17], [18], [19], but their protective role against VL is still to decipher. It was also revealed from previous studies carried out in cutaneous and visceral leishmaniasis patients, that the humoral response induced was found to be specific for *Leishmania* histones since no cross reactivity of VCL sera with mammalian histones were observed [20]. In the present study cloning, expression, purification and molecular characterization of these four histone proteins of *L. donovani* was reported for the first time. The ability of recombinant rLdH2B, rLdH3, rLdH2A and rLdH4, either individually or in the pooled form (rLdH2-4) was also assessed to stimulate the immune responses in PBMCs/lymphocytes of *leishmania* infected cured/endemic contact individuals as well as hamsters. In addition to it the protective efficacy of individual histones and their combinations was measured in naive hamsters against *L. donovani* challenge.

## Materials and Methods

### Ethical statement

All animal care and experimental use conformed to Committee for the Purpose of Control and Supervision on Experiments on Animals guidelines for laboratory animal facilities and were approved by the Committee on the Ethics of Animal Experiments of Central Drug Research Institute (protocol number IAEC/2009/Para/117). The protocol and study with patients' was approved by the Ethics committee of the Kala-azar Medical Research Centre, Muzaffarpur (Protocol # EC-KAMRC/Vaccine/VL/2007-01) and written informed consent was obtained from patients before enrollment in this study. All the human subjects underwent clinical examination by a local physician for leishmanial and other possible infections.

### Animal

Laboratory-bred male golden hamsters (Mesocricetus auratus, 45–50 g) from the Institute's animal house facility were used as experimental host. They were housed in climatically controlled room and fed with standard rodent food pellet (Lipton India Ltd., Bombay) and water ad libitum. The experimental animals were monitored daily till the termination of the study and were autopsied at that time point or whenever required under the deep anesthesia with over dosages of sodium thiopentone in order to reduce the suffering level in experimental animals. Thiopentone was injected into animals with saline solution at a dose of 50 mg/kg of the body weight through intraperitoneally route.

### Parasites Culture

The *L. donovani* WHO reference strain Dd8 (MHOM/In/80/Dd8) has been cultured *in vitro* as described elsewhere (21). For bulk amount, promastigotes were cultured in RPMI-1640 medium supplemented with 10% heat-inactivated fetal bovine serum (Sigma, USA), 100 U/ml penicillin (Sigma, USA) and 100 µg/ml streptomycin (Sigma, USA) at 26°C±1°C for 3–5 days 75 cm^2^ culture flasks (Corning). The strain has also been maintained *in vitro* as well as *in vivo* in hamsters through serial passages, i.e. from amastigote to amastigote [21].

### Cell line

Mouse macrophage cell line J774A.1 was procured from Tissue culture Facility of the institute and maintained in RPMI-1640 through serial passage in 75 cm^2^ culture flasks (Corning) at 37°C and 5% CO_2_. The confluent cells were harvested using cell scrapper for the estimation of nitric oxide (NO) production.

### Preparation of soluble *L. donovani* promastigote antigen (SLD)

SLD was prepared as per method described by [22]. Briefly, stationary phase promastigotes (10^9^) were harvested from 3 to 4 days of culture and washed four times in cold PBS, resuspended in PBS containing protease inhibitor cocktail (Sigma, USA) and subjected to ultrasonication and higher range of centrifugation at 40,000×g for 30 min. The protein content of the supernatant was estimated and stored at −70°C for the use of longer time.

### Cloning, expression and purification of *L. donovani* histone proteins (LdH2B, LdH3, LdH2A, LdH4)

Genomic DNA was isolated from 10**^8^** *L. donovani* promastigotes, washed and suspended in NET buffer (10 mM Tris-HCl (pH 7.5), 100 mM NaCl, and 1 mM EDTA) and incubated with proteinase K (1 mg/ml; Invitrogen Life Technologies) and 0.5% SDS at 50°C for 3 h. Nucleic acids were extracted by phenol: chloroform: isoamyl alcohol extraction and ethanol precipitation. Genomic DNA was spooled and subjected to RNase (100 µg/ml) treatment. LdH2B, LdH3, LdH2A and LdH4 gene were amplified using Ldhistone-specific primers which were designed as mentioned in Table1 on the basis of *L.infantum* histone - LdH2B, LdH3, LdH2A, LdH4 genes sequences available at National Center for Biotechnology Information (NCBI). PCR was performed using Taq Polymerase (Fermentas) lacking a 3′–5′ exonuclease activity in a Thermocycler (Bio-Rad) under conditions at one cycle of 95°C for 3 min, 30 cycles of 95°C for 1 min, 55°C for 1 min, and 72°C for 2 min, and finally one incubation at 72°C for 10 min. Amplified PCR product was electrophoresed in agarose gel and eluted by using Gen Elute Columns (Qiagen). Eluted product was ligated in pTZ57R/T (T/A) cloning vector (Fermentas) and transformed into competent *Escherichia coli DH5α* cells. The transformants were screened for the presence of recombinant plasmids with the rLdH2B, rLdH3, rLdH2A, rLdH4 genes insert by gene-specific PCR under similar conditions as previously mentioned. Isolated positive clones were sequenced (from South Campus, Delhi University, NewDelhi, India) and later submitted to the NCBI.

**Table 1:** Sequences of forward and reverse primers of Histone gene.

S.No	Gene	Forward Primer	Reverse Primer
1	H2B	5′GGATCCATGTCGCGCACCAAGGAGACC-3′	5′GAATTCGTCCCCCTCACCGCGCAG-3′
2	H3	5′GGATCCATGGCCTCTTCTCACTCTGCTTC-3′	5′GAATTCACGCGACGGGCACGACACGGC-3′
3	H2A	5′-GAATTCACGCGACGGGCACGACACGGC-3′	5′GAATTCTGCGCTCGG TGTCGCCTTGCC-3′
4	H4	5′-GGATCCATGGCTACTCCTCGCAGCGC-3′	5′-GAATTCCGCGTAGCCGTACAGGATGTG-3′

(http://www.ncbi.nlm.nih.gov/nuccore/GU066394/HM057222/JF78400/GQ845113).

(Accession nos. GU066394/HM057222/JF78400/GQ845113). rLdH2B, rLdH3, rLdH2A, rLdH4 were further subcloned at the *BamHI* and *EcoRI* site in bacterial expression vector- pET28a**^+^** (Novagen).Their expressions were checked in bacterial cells by transforming the rLdH2B+ pET28a**^+^**/rLdH3+ pET28a**^+^**/rLdH2A+ pET28a**^+^**/rLdH4 + pET28a**^+^** constructs in *E. coli Rosetta* strain separately. The transformed cells were inoculated in to 5 ml Luria Bertani medium (LB) containing 34 µg/mL of chloramphenicol and 35 µg/mL kanamycin and allowed to grow at 37°C with shaking at 200 rpm. Cultures in logarithmic phase (at OD 600 of ∼0.5–0.6) were induced for 16 h with 1.0 mM isopropyl-b-D-thiogalactopyranoside (IPTG) at 20°C. After induction, 1 ml cells were lysed in 100 µl sample buffer (50 m M Tris-HCl (pH 8), 10% SDS, and 0.05% bromophenol blue, with 100 mM DTT) and whole cell lysates (WCL) were analyzed by 12% SDS-PAGE. Uninduced control cultures were analyzed in parallel. The overexpression of rLdH2B, rLdH3, rLdH2A and rLdH4 was visualized by staining the gel with Coomassie brilliant blue R-250 (Sigma).

For purification of each histone protein, 200 mL of LB medium induced as mentioned above and processed by affinity chromatography using Ni**^2+^**chelating resin to bind the His6-tag fusion peptide derived from the pET28a**^+^** vector. The cell pellets were resuspended separately in 4 mL of lysis buffer (50 mM Tris-HCL (pH 8.0), 300 mM NaCl) containing 1∶200 dilution of protease cocktail inhibitor (Sigma) and 1% Triton X-100, incubated for 30 min on ice with 1 mg/mL lysozyme (Sigma), and the suspension was sonicated for 10×20 sec (with 30 s intervals between each pulse) on ice. The sonicated cells were centrifuged at 15,000 g for 30 min at 4°C, and the supernatant was incubated at 4°C for 1 h with the 2 mL of Ni–NTA Superflow resin (Qiagen, Hilden, Germany) previously equilibrated with lysis buffer. After washing with buffer (50 mM Tris-HCL (pH 8.0), 300 mM NaCl), containing different concentrations of imidazole i.e. 20,50 and 100 mM, each of the purified recombinant protein was eluted with elution buffer (50 mM Tris-HCL, 300 mM NaCl, and 250 mM imidazole, pH 7.5). The eluted fractions were analyzed by 12% SDS–PAGE followed by Coomassie blue staining. The protein content of the fractions was estimated by the Bradford method using BSA as standard.

### Production of polyclonal antibodies against rLdH2B, rLdH3, rLdH2A, rLdH4 and Western blot analysis

The purified rLdH2B, rLdH3, rLdH2A and rLdH4 proteins were used for raising antibodies in Swiss mice. Mice were first immunized using 50 µg of each of the recombinant histone proteins in Freund's complete adjuvant. After 15 days, the mice were given 3 booster doses of 25 µg of the protein in incomplete Freund's adjuvant at 2-weeks interval and blood was collected for serum 8 days after the last immunization. Antibody titre was determined by ELISA and was found to be 1∶64000. For immunoblotting experiment, purified r-histone protein along with SLD were resolved in 12% SDS–PAGE and transferred to nitrocellulose membrane using a semi-dry blot apparatus (Amersham) [23]. After overnight blocking in 5% BSA, the membrane was incubated with antiserum to the r-histone protein at a dilution of 1∶2000 for 120 min at room temperature (RT). The membrane was washed three times with PBS containing 0.5% Tween 20 (PBS-T) and then incubated with secondary antibody - goat anti-mice IgG HRP conjugate (Bangalore Genie) at a dilution of 1∶10,000 for 1 h at RT. The blot was developed by using diaminobenzidine hydrochloride + imidazole +H_2_O_2_ (Sigma).

### Patients and isolation of PBMCs

The study groups of human samples were as follows:

[1] Six treated cured patients (3 males and 3females, age ranging from 15–50 years) from hyper-endemic areas of Bihar. All the patients had received a complete course of amphotericin B and had recovered from VL. Samples were collected for 2 months to 1 year after the completion of treatment. The diagnosis was established in all cases by demonstration of parasites in splenic aspirates and found negative at the time of study.

[2] Six endemic household contacts (2 males and 4 females, age range-25 to 45 years) that have neither showed clinical symptoms nor received any treatment for Kala-azar. They belonged to the family of infected/cured patients.

[3] Six infected patients (3 males and 3 females, age range- 10 to 55 years) showing clinical symptoms of Kala-azar.

[4] Six normal healthy donors (2 males and 4 females, age range 10–50 years) from non-endemic areas, without any history of leishmaniasis, served as negative control.

Heparinized venous blood (10 ml each) was collected from all the study subjects and peripheral blood mononuclear cells (PBMCs) were isolated from blood by Ficoll-Hypaque density gradient centrifugation (Histopaque 1077, Sigma, USA) as described by [21]. A final suspension of 1×10^6^ cells/ml was made in complete RPMI medium (cRPMI) after determining cell viability by trypan blue staining method. These were used for various immunological assays.

### Treatment of *L. donovani* infected hamsters and isolation of mononuclear cells (lymph node cells)

Approximately 20 hamsters, infected with 10**^7^** amastigotes intracardially, were assessed one month later for parasitic burden by splenic biopsy through a small incision in the upper left quarter of the abdomen and a small piece of splenic tissue was cut and dab smears were made on slides. The incised portion was stitched with nylon suturing thread. Following biopsy, an adequate amount of antibiotic powder (Neosporin) was applied to the stitched portion and finally sealed with tincture of benzoin. In addition, neosporin sulphate (100 mg/kg of body weight) was also given orally the day before and the day after the biopsy for healing. The smears were fixed in methanol, stained with Giemsa and the number of amastigotes/1000 cell nuclei was counted. The animals harbouring >25–30 amastigotes/100 macrophage cell nuclei were then treated with antileishmanial drug-Miltefosine (Zentaris, Germany) at 40 mg/kg bodyweight daily for 5 days. The animals were reassessed for complete cure by splenic biopsy performed on day 30 post-treatment. The animals were then autopsied as described above and mononuclear cells were separated from inguinal and mesenteric lymph nodes lymph nodes of cured, infected as well as normal hamsters following the protocol of Garg et al [14] and a suspension of 10^6^ cells/ml was made in cRPMI. These cells were employed for lymphoproliferative assay and in the estimation of NO production.

### Immunological assays

#### Assessment of Lymphocyte proliferative responses (LTT) in cured/exposed patients and hamsters

Lymphocytes suspension (1×10^6^ cells/ml) of cured/exposed patients and normal, infected (30 days p.i.) and cured hamsters was cultured in 96-well flat bottom tissue culture plates (Nunc, Denmark). This assay was carried out as per protocol described by [14] with some modifications, wherein XTT (Roche diagnostics) was used instead of 3H thymidine. About 100 µl of a predetermined concentration (10 µg/ml) of mitogens- PHA for Patient's PBMCs and ConA for hamster's lymphocytes were added to the wells in triplicate. Similarly, PBMCs were stimulated for 5 days with recombinant histones at a concentration of 10 µg/ml (10 µg/ml of an individual histone protein or 2.5 µg/ml of each of the four recombinant histones in the combination) SLD served as positive control at a concentration of 10 µg/ml. Wells without stimulants served as blank controls. Cultures were incubated at 37°C in a CO_2_ incubator with 5% CO**_2_** for 3 days in the case of the mitogens, and for 5 days in the case of the antigens. Eighteen hours prior to termination of culture, 50 µl of XTT was added to 100 µl of supernatants of each well and the absorbance measured at 480 nm with 650 nm as reference wavelength.

#### Estimation of NO activity in macrophages of cell lines

Isolated lymphocytes from normal, infected (day 30 p.i.) and cured (day 30 p.t) group of hamsters were suspended in culture medium and plated at 10^5^ cells/well and stimulated for 3 days in case of mitogen (LPS) and 5 days in case of recombinant antigens (either individually or in pooled form as stated above) and SLD at 10 µg/ml. The presence of NO was assessed using Griess reagent (Sigma, U.S.A) in the culture supernatants of macrophages of cell lines (J774 A.1) after the exposure with the supernatant of stimulated lymphocytes. The supernatants (100 µl), collected from macrophage cultures 24 h after incubation, was mixed with an equal volume of Griess reagent and left for 10 min at room temperature. The absorbance of the reaction was measured at 540 nm in an ELISA reader [24]. The nitrite concentration in the macrophage culture supernatant samples was extrapolated from the standard curve plotted with sodium nitrite.

#### Assessment of Cytokine levels- IFN-γ/IL-12/IL-10 in lymphocytes of cured/endemic patients

Culture of PBMCs (1×10^6^ cells/ml) from human patients was set up in 96-well culture plates and recombinant histone proteins viz. rLdH2B, rLdH3, rLdH2A and rLdH4 were taken in the concentration of 10 µg/ml either individually or in pooled form (rLdH2-4) was added in a triplicate wells. The level of IFN-γ, IL-12 as well as IL-10 was estimated by ELISA kit (OptEIA set, Pharmingen) after 5 days of incubation with antigens using supernatants. The results were expressed as picograms of cytokine/ml, based on the standard curves of the respective cytokine provided in the kit.

#### Measurement of antibody response in hamsters

The level of IgG antibody and its isotypes in sera samples of hamsters of different experimental groups was measured as per protocol by [25] with slight modifications. Briefly, 96-well ELISA plates (corning) were coated with 0.2 µg/100 µl/well recombinant histone proteins (individually or in pooled form) overnight at 4°C and blocked with 1.5% BSA at room temperature for 1 h. Sera was used at a dilution of 1/100 for IgG, IgG1, and IgG2 and kept for 2 h at RT. Biotin-conjugated mouse anti-Armenian and Syrian hamster IgG, IgG1 and biotinylated anti-Syrian hamster IgG2 (BD Pharmingen) were added for 1 h at room temperature at 1/1000 dilutions and were further incubated with peroxidase-conjugated streptavidin at 1/1000 (BD Pharmingen) for 1 h. Finally, the substrate O-phenylenediamine dihydrochloride (Sigma) was added and the plate was read at 492 nm.

### Prophylactic efficacy of recombinant Histone protein(s) with or without BCG

Four groups of hamsters containing 12–15 animals per group, were taken for the study, wherein groups 1–3 served as controls i.e.- group 1- unvaccinated and unchallenged (normal control); group 2- unvaccinated and challenged (infected control) and group 3-BCG alone. The animals of group 4 were immunized intradermally on the back with a dose of 50 µg/50 µl of either recombinant proteins rLdH2B/rLdH3/rLdH2A/rLdH4 along with equal volume of 0.1mg per animal BCG. Similarly, the animals were also immunized with recombinant histone proteins pooled (rLdH2-4+BCG) in emulsified form. Fifteen days later a booster dose of half of the amount of recombinant histone proteins either individually or in the pooled form along with BCG was given intradermally to all the hamsters of Group 4 and only BCG to the animals of group 3. Twenty one days later the hamsters of Groups 2–4 were challenged intracardially with 10^7^ metacyclic promastigotes of *L. donovani*. Three to four animals from each group were sacrificed as described above on days 45, 60, 90 and 120 p.c., for the assessment of parasitological and immunological (cytokines by real-time PCR and Antibody level by ELISA and lymphoproliferation and NO production by above mention methods) progression of VL. The impression smears/touch blots of different organs, namely spleen, liver, and bone marrow (femur bone), of experimental animals were made, and the criteria for the assessment of parasite burden was based on the counting of the number of amastigotes per 100 cell nuclei in each organ. The percentage of inhibition of parasite multiplication was calculated in comparison with the unvaccinated control using the following formula: percentage of inhibition  =  (number of parasite count from infected control - number of parasites from the vaccinated group/number of parasite count from infected control) ×100. Animal of all the groups were given proper care and observed for their survival period.

#### Quantification of mRNA cytokines and inducible NO synthase (iNOS) in hamsters by Real time-PCR

qRT-PCR was performed to assess the expression of mRNAs for various cytokines and iNOS in splenic cells. Splenic tissues were taken from each of the three randomly chosen animals. Total RNA was isolated using Tri-reagent (Sigma-Aldrich) and quantified by using Gene-quant (Bio-Rad). One microgram of total RNA was used for the synthesis of cDNA using a first-strand cDNA synthesis kit (Fermentas). For real-time PCR, primers were designed using Beacon Designer software (Bio-Rad) on the basis of cytokines and iNOS mRNA sequences available on PubMed (4) (Table-2). qRT-PCR was conducted as per the protocol described earlier [26] by using 12.5 µl of SYBR green PCR master mix (Bio-Rad), 1 µg of cDNA, and primers at a final concentration of 300 nM in a final volume of 25 µl. PCR was conducted under the following conditions: initial denaturation at 95°C for 2 min followed by 40 cycles, each consisting of denaturation at 95°C for 30 s, annealing at 55°C for 40 s, and extension at 72°C for 40 s per cycle using the iQ5 multicolor real-time PCR system (Bio-Rad). cDNAs from normal hamsters were used as “comparator samples” for quantification of those corresponding to test samples whereas in vaccination studies, cDNAs from infected hamsters were used as “comparator samples”. All quantifications were normalized to the housekeeping gene HPRT. A no-template control c-DNA was included to eliminate contaminations or nonspecific reactions. The cycle threshold (CT) value was defined as the number of PCR cycles required for the fluorescence signal to exceed the detection threshold value (background noise). Differences in gene expression were calculated by the comparative CT method [25]. This method compares test samples to a comparator sample and uses results obtained with a uniformly expressed control gene (HPRT) to correct for differences in the amounts of RNA present in the two samples being compared to generate a ΔCT value. Results are expressed as the degrees of difference between ΔCT values of test and comparator samples.

**Table 2:** Sequences of forward and reverse primers of hamster cytokines used for quantitative real time RT-PCR.

S.N.	Primer	Primer sequence
1.	HGPRT	Forward 5′ GATAGATCCACTCCCATAACTG 3′
		Reverse 5′ TACCTTCAACAATCAAGACATTC 3′
2.	TNF-α	Forward 5′ TTCTCCTTCCTGCTTGTG3′
		Reverse 5′ CTGAGTGTGAGTGTCTGG3′
3.	IFN-γ	Forward 5′ GCTTAGATGTCGTGAATGG 3′
		Reverse 5′ GCTGCTGTTGAAGAAGTTAG 3′
4.	IL-12	Forward 5′ TATGTTGTAGAGGTGGACTG3′
		Reverse 5′ TTGTGGCAGGTGTATTGG 3′
5.	TGF-β	Forward 5′ ACGGAGAAGAACTGCTGTG 3′
		Reverse 5′ GGTTGTGTTGGTTGTAGAGG 3′
6.	IL-4	Forward 5′ GCCATCCTGCTCTGCCTTC 3′
		Reverse 5′ TCCGTGGAGTTCTTCCTTGC 3′
7.	IL-10	Forward 5′ TGCCAAACCTTATCAGAAATG3′
		Reverse 5′ AGTTATCCTTCACCTGTTCC 3′
8.	iNOS	Forward 5′ CGACGGCACCATCAGAGG 3′
		Reverse 5′AGGATCAGAGGCAGCACATC 3′

#### Measurement of Antibody response in vaccinated/immunized hamsters

The level of IgG antibody and its isotypes in serum samples of hamsters of different experimental groups was measured as per protocol by [25] with slight modifications. Briefly, 96-well ELISA plates (corning) were coated with rLdhistones (0.2 µg/100 µl/well) overnight at 4°C and blocked with 1.5% BSA at room temperature for 1 h. Sera was used at a dilution of 1/100 for IgG, IgG1, and IgG2 and kept for 2 h at RT. Biotin-conjugated mouse anti-Armenian and Syrian hamster IgG, IgG1 and biotinylated anti-Syrian hamster IgG2 (BD Pharmingen) were added for 1 h at room temperature at 1/1000 dilutions and were further incubated with peroxidase-conjugated streptavidin at 1/1000 (BD Pharmingen) for 1 h. Finally, the substrate O-phenylenediamine dihydrochloride (Sigma) was added and the plate was read at 492 nm.

### Statistical analysis

Results were expressed as mean±S.D. Two sets of experiments were performed and the results represent the pooled data of the two experiments. Results were analyzed by student t test and one-way ANOVA test followed by Dunnets or Tukeys post which ever appropriate at each case using Prism Graphpad software program. The upper level of significance was chosen as p<0.001 (highly significant).

## Results

**Figure 1:**
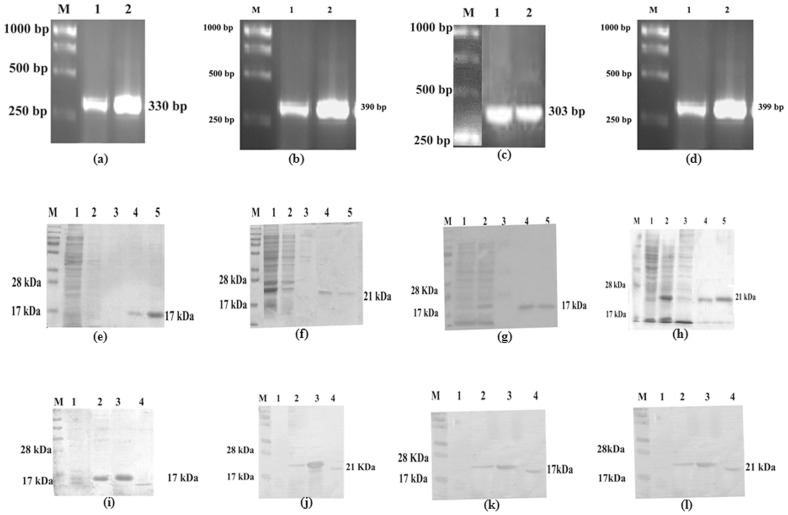
(a,b,c & d)- PCR of of LdH2B, LdH3, LdH4 and LdH2A genes. (e, f, g, & h)- Expression and purification of LdH2B, LdH3, LdH2A, LdH4 from *E. coli* rossetta cells, WCL of transformed *E. coli* separated on 12% acrylamide gel and stained with Coomassie blue, M: Molecular wt. markers; Lane 1: Flow through; lane 2&3: Washing fraction; Lane 4 & 5: eluted protein. (i, j, k & l)-Western blot analysis using anti-rLhistone antibody in uninduced WCL, induced WCL and *leishmania* WCL - M: Mol wt marker, Lane 1: uninduced WCL, Lane 2: induced WCL, Lane 3: Purified protein, Lane 4: whole cell lysate of *Leishmania*.

All the four histone proteins of *L.donovani* - LdH2B, LdH3, LdH2A and LdH4 were successfully cloned, sequenced and expressed in *E. coli Rosetta* strain (Fig1.a, b, c & d). H3 and H4 exhibited ∼90% homology with histones of *L.major* and *L.infantum* whereas the other two H2A and H2B were homologous to the tune of 82-85%. It was further sub-cloned in bacterial expression vector pET28a^+^ and was eluted at 250 mM imidazole concentration (Fig1.e, f, g & h). Immunoblots of lysates from *L. donovani* promastigote was performed with the polyclonal antibodies raised against each of rLdH2B, rLdH3, rLdH2A and rLdH4 respectively, which detected one dominant protein band of ∼17kDa, ∼21kDa, ∼17kDa and ∼21kDa respectively (Fig1. i, j, k & l). The presence of the lipopolysaccharides (LPS) content of the recombinant proteins was measured by Limulus amoebocyte lysate test (QCL-1000, Lonza, Walkersville, MD. USA) and was found to be below 10 endotoxin units (EU)/mg of the recombinant protein.

### Recombinant histone proteins induced significant lymphoproliferative and NO responses in normal/infected/cured hamsters

**Figure 2:**
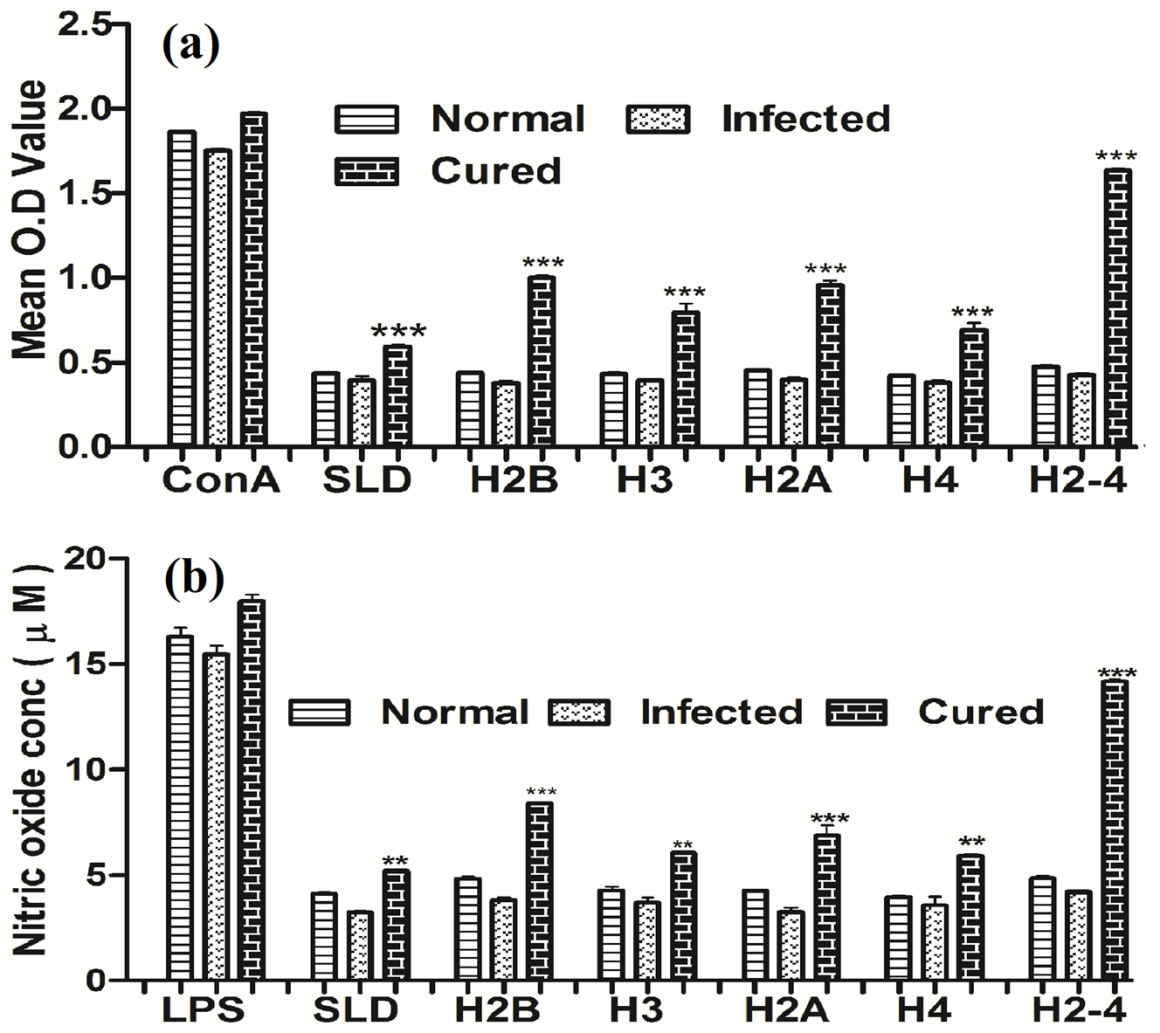
Cellular responses of rLdH2B, rLdH3, rLdH2A, rLdH4, and pooled(H2-4) of *L.donovani* in hamsters. **(a)** XTT response of mononuclear cells (lymph nodes) from normal, *L.donovani* infected (30 day p.c.) and treated hamsters in response to Con A, SLD and rLdH2B, rLdH3, rLdH2A, rLdH4 and pooled (rLdH2-4) at 10 µg/ml each. Proliferation was represented as mean OD of stimulated culture-mean OD of unstimulated control. Each bar represents the pooled data (mean ± S.D. value) of 6 hamsters and the data represent the means of triplicate wells ± the S.D. **(b)** Nitric oxide production (µM) by the J774 A.1 cells were primed with the supernatants of stimulated lymphocytes (3 days with mitogen and 5 days with antigens) of normal, infected and cured hamsters in response to rLdH2B, rLdH3, rLdH2A, rLdH4 and pooled (H2-4), SLD and LPS respectively at 10 µg/ml each. The estimation of NO production was done using Greiss reagent in supernatants collected from macrophage cultures 24 h after incubation and the absorbance of the reaction product was measured at 540 nm. Significance values indicate the difference between the SLD and rLdH2B, rLdH3, rLdH2A, rLdH4, and pooled (H2-4) stimulation (**, p<0.01; ***, p<0.001).

The cellular responses of lymph node cells of cured hamsters were assessed by XTT against the mitogen - Con A as well as antigens- SLD, individual recombinant histone proteins – viz. rLdH2B, rLdH3, rLdH2A and rLdH4. Simultaneously cellular responses against the pooled combination of all the four recombinant histone proteins -rLdH2-4 were also assessed. The responses were compared with that of normal as well as *L. donovani* infected groups that served as controls. In general, the normal and infected controls as well as cured *Leishmania* infected group had shown higher proliferative responses against ConA (Fig 2(a)). The lymphoproliferative response against all the individual proteins was moderately higher (1.5 to 2.5 fold) in cured hamsters as compared to *L. donovani* infected hamster. However, an optimum stimulation to the tune of 4 folds was observed when all the four proteins were pooled (rLdH2-4) which was highly significant (P<0.001).

NO-mediated macrophage effector mechanism is known to be critical in the control of parasite replication in the animal model hence its production in macrophages of cell line J774 A.1, was studied after 24 h of incubation in the presence of individual rLdH2B, rLdH3, rLdH2A and rLdH4 proteins as well as pooled (rLdH2-4). For comparison, NO production in mitogenic (LPS) stimulated and unstimulated cells served as positive as well as negative controls respectively (Fig. 2 (b). Among the four individual histone proteins, NO production was recorded to be optimum against rLdH2B and rLdH2A whereas the rLdH2-4 yielded maximum NO production (p<0.001).

### rLdH2-4 stimulates PBMCs from *Leishmania* Patients optimally to proliferate and to express a Predominant Thl Cytokine Profile

**Figure 3:**
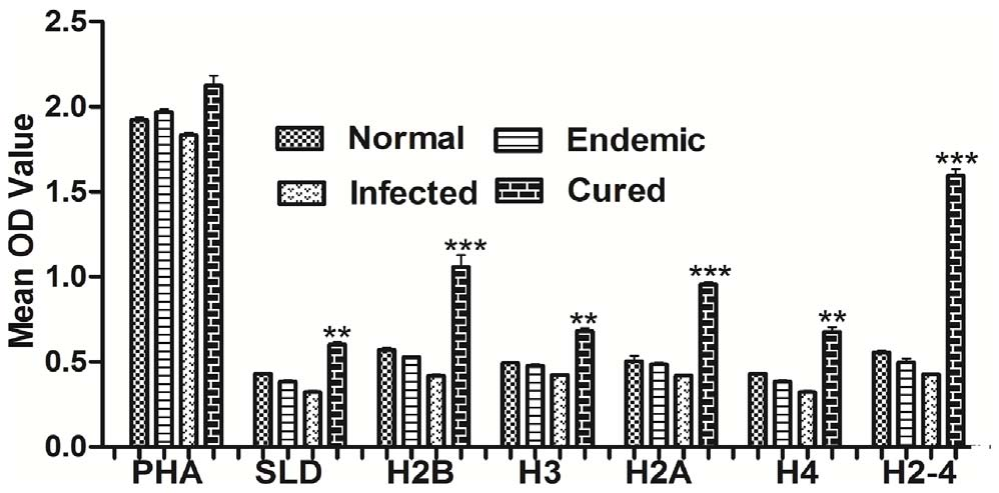
Cellular responses of rLdH2B, rLdH3, rLdH2A, rLdH4, and pooled (rLdH2-4) of *L.donovani* in patients sample. : XTT response of PBMC from normal, *L. donovani* infected, Endemic and cured patients in response to PHA, SLD and rLdH2B, rLdH3, rLdH2A, rLdH4 and pooled (H2-4) at 10 µg/ml each. Proliferation was represented as mean OD of stimulated culture - mean OD of unstimulated control. Each bar represents the data (mean ± S.D. value) of triplicate wells ± the S.D. pooled (rLdH2-4) shown significantly higher cellular response. Significance values indicate the difference between the SLD and rLdH2B, rLdH3, rLdH2A, rLdH4, and pooled (rLdH2-4) stimulation (**, p<0.01; ***, p<0.001).

Further, the cellular responses (XTT and cytokine levels) of all the four recombinant histone proteins individually as well as their combination were validated in PBMCs of cured patients, endemic and non-endemic controls and *L.donovani*-infected donors. Individual donors in each study group were found to elicit different responses. PBMCs of all groups exhibited higher proliferation against PHA as compared to unstimulated control. Significantly higher proliferative response from all the cured patients was observed against rLdH2B, rLdH3, rLdH2A and rLdH4 as well as their pooled (rLdH2-4) with mean OD values being 1.2 to 2.6 folds higher than SLD (Fig.3). The results demonstrated that pooled (rLdH2-4) was the potent immunogens recognized by a majority of *L.donovani* –infected/cured/endemic individuals at different stages or manifestations of infection.

**Figure 4:**
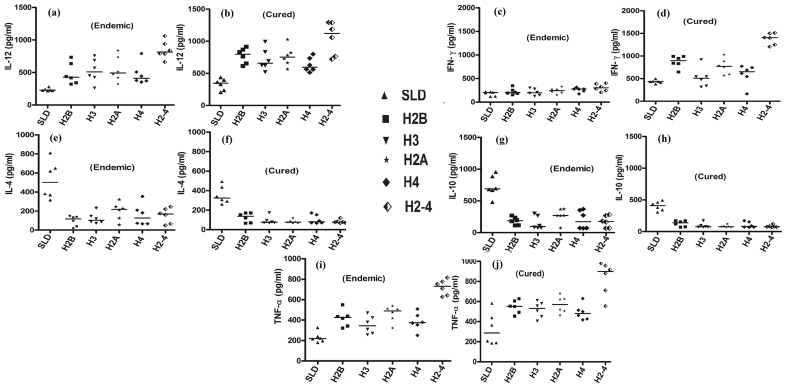
Th1 and Th2 cytokine production in PBMCs from individuals of cured VL patients (6), infected individuals (6) and endemic controls (6). , in response to rLdH2B, rLdH3, rLdH2A, rLdH4 and their combination and mix and SLD antigens, each data point represents one individual. Values are given as concentration in pg/ml. Significance values indicate the difference between the SLD and rLdH2B, rLdH3, rLdH2A, rLdH4 and their combination and mix stimulation of PBMCs (**, p<0.01; ***, p<0.001; and ***, p<0.001).

To determine the Th1/Th2 stimulatory potential of the rLdHistones and pooled combination (rLdH2-4) we further assessed the cytokine levels viz. IFN-γ, TNF-α, IL-12 as well as IL-10 and IL-4 in PBMCs from cured patients as well as in endemic contacts. The levels of IFN-γ and TNF-α against pooled (rLdH2-4) histone protein was observed to be 1.5 to 2 fold higher as compared to individual histones in the supernatants of cured patients (Fig.4) On the contrary, very low levels of IL-10 and IL-4 cytokines against individual rLdHistone proteins and pooled (rLdH2-4) were detected in supernatants of cured patients followed by endemic contacts (Fig.4). PBMCs of cured/endemic contacts generated a mixed ThI/Th2 cytokine profile against SLD wherein higher levels of IL-10/IL-4 and lower level of IFN-γ and IL-12 were noticed in response to SLD in cured as well as endemic contacts.

### rLdH2B, rLdH3, rLdH2A and rLdH4 modulates *Leishmania*-specific IgG and its isotypes in naïve hamsters as well as in cured and endemic patient sample

**Figure 5:**
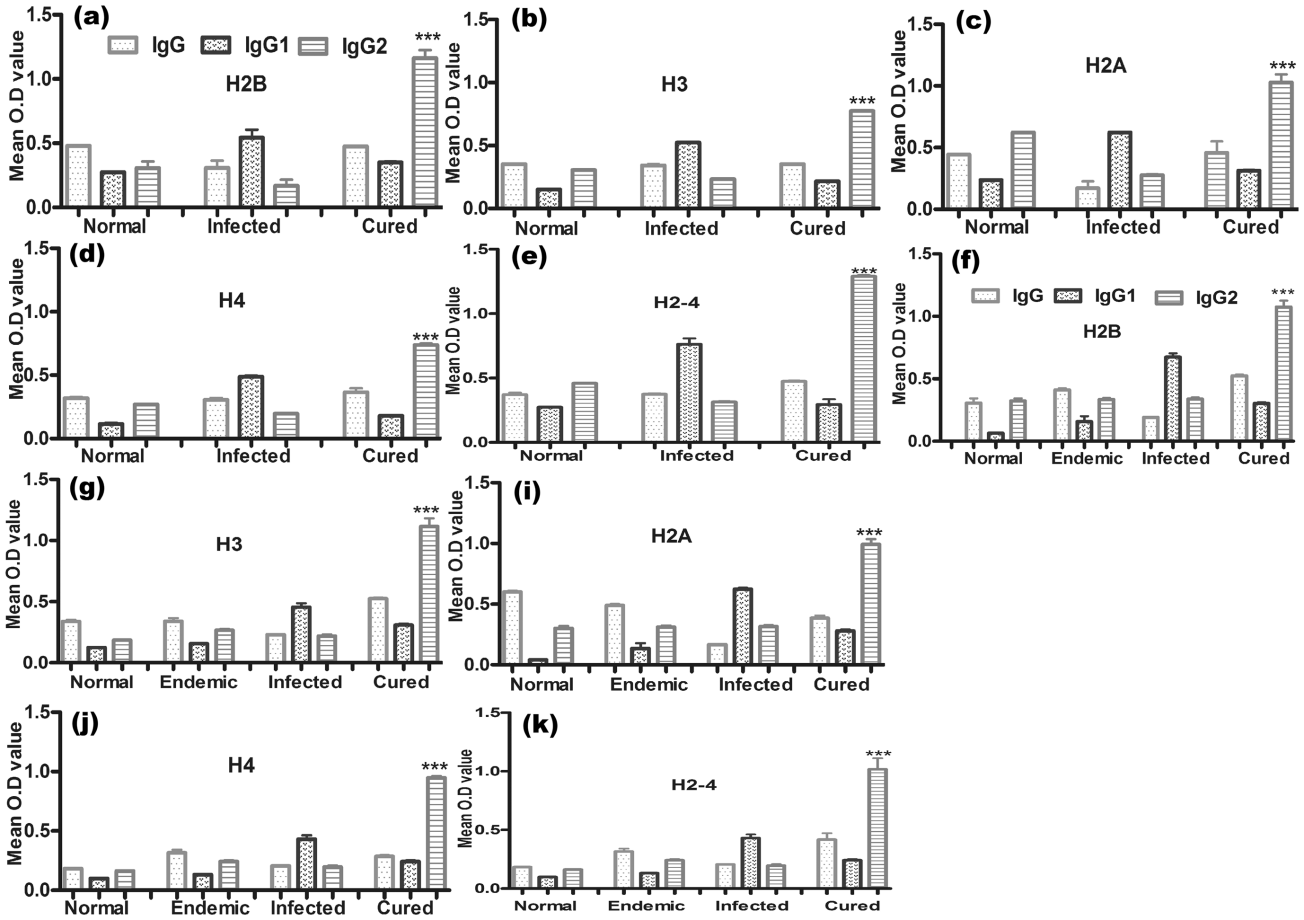
Antibody response shown (OD value) in hamster's serum with rLdH2B, rLdH3, rLdH2A, rLdH4 and pooled (H2-4). (a, b, c, d, e) and in patient sample (f, g, h, i, j). Serum samples were collected from different groups of hamsters at designated time points and assayed for specific IgG, IgG1, and IgG2 levels by ELISA. Each bar represents the pooled data (mean±S.D. value) of three replicates. Significance values indicate the difference between the cured group and normal group (***, p<0.001).

To assess the antibody level in the serum of cured animals, we further estimated the level of IgG and its isotypes in response to all the recombinant histone proteins and their pooled (rLdH2-4). In general, we observed significantly higher (∼3 to 4 fold) IgG2 response in cured animals as compared to the normal and infected animals (Fig.5) against pooled (rLdH2-4) in comparison to the each histone proteins. But there was no apparent difference in the IgG1 response between the cured and the normal animals against any of them.

Further, the level of IgG and its isotypes in cured/infected *Leishmania* patients were validated. Significantly higher (∼2.5 to 3 fold) IgG2 response was observed by cured patients as compared to the endemic contacts and infected patients. However, there was no apparent difference in the IgG1 response between the endemic and the cured patients (Fig.5).

### Immunization with recombinant LdH2-4+BCG induced optimum prophylactic efficacy against *L. donovani* challenges

**Figure 6:**
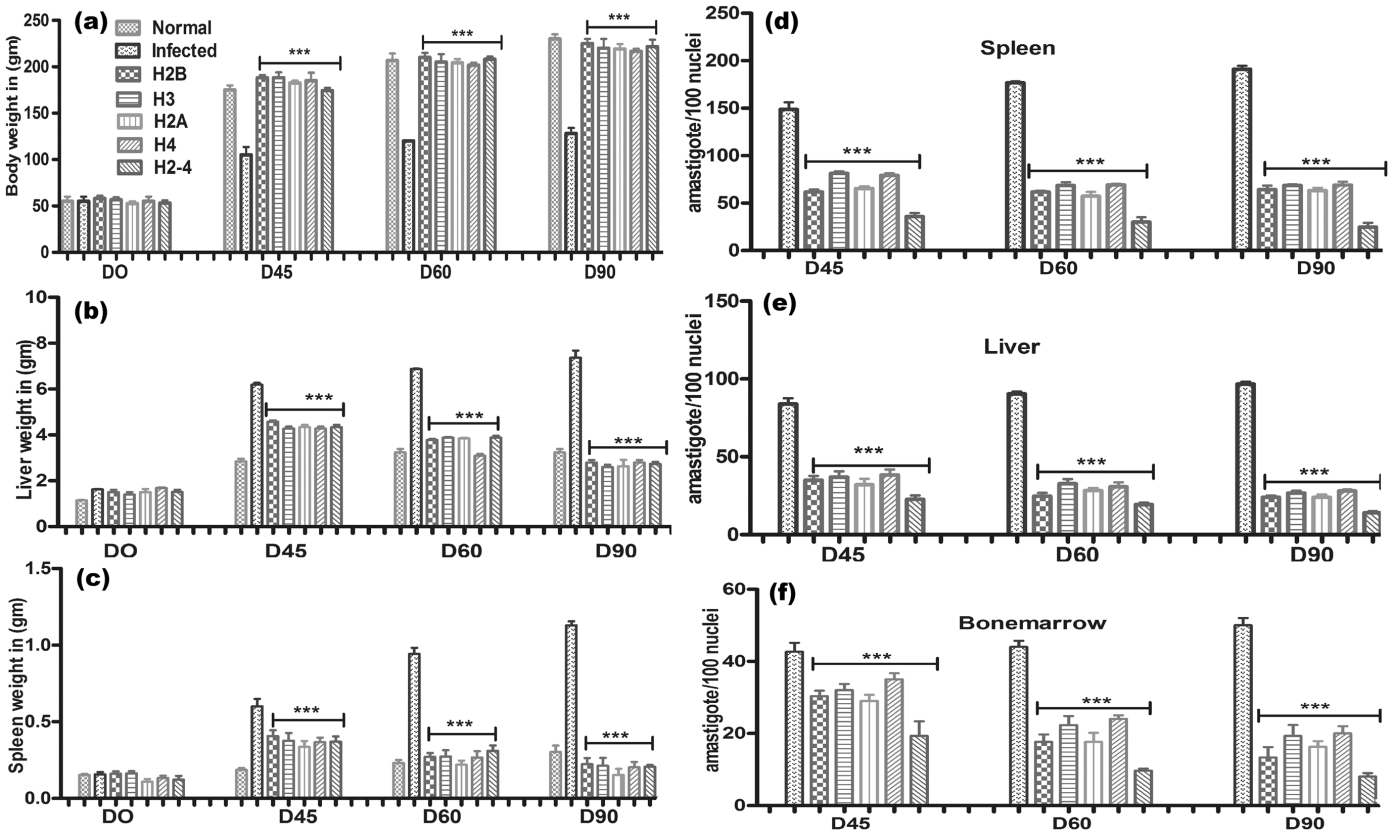
Clinical outcomes following *L. donovani* challenge in hamsters immunized with rLdH2B+BCG, rLdH3+BCG, rLdH2A+BCG, rLdH4+BCG and pooled (H2-4 +BCG). On day 21 after the booster, the hamsters of infected, BCG alone and vaccinated groups were challenged intracardially with 10^7^ metacyclic promastigotes of *L. donovani*. Following parameters observed Body weight (a), spleen weight (b), liver weight (c) and Parasite burden (no. of amastigotes per 100 cell nuclei) in the spleen (d), liver (e), and bone marrow (f) on days 45, 60, 90 p.c. Data represent mean values standard errors (SE) at the designated time points in the experiments. The hamsters immunized with rLdH2-4+BCG and challenged with *L. donovani* exhibited an optimal reduction in parasite load in spleen, liver and bone marrow Significance values indicate the difference between the vaccinated groups and infected group (***, p<0.001).

The hamsters immunized with recombinant histone protein(s) either individually or in collective forms alongwith BCG, in general, gained considerable weight as compared to those groups of hamsters which were immunized with BCG alone as well as to unimmunized infected animals, when kept simultaneously upto day 90 post challenge (p.c). The hamsters immunized with rLdH2-4+BCG and challenged with *L. donovani* exhibited an optimal reduction in parasite load (∼80%) in spleen, liver and bone marrow on day 90 p.c which was significantly higher (p<.0001) than all the other experimental as well control groups. Lesser efficacy (∼55%) was obtained in hamsters immunized with individual rLdH2B/rLdH3/rLdH2A/rLdH4 alongwith BCG. The survival of rLdH2-4+BCG immunized hamsters after the *Leishmania* challenge was the longest (6 months post-infection) as compared to the unimmunized ones (day 60 to 90 p.c.) wherein progressive increase in parasite load was observed (Fig. 6).

**Figure 7:**
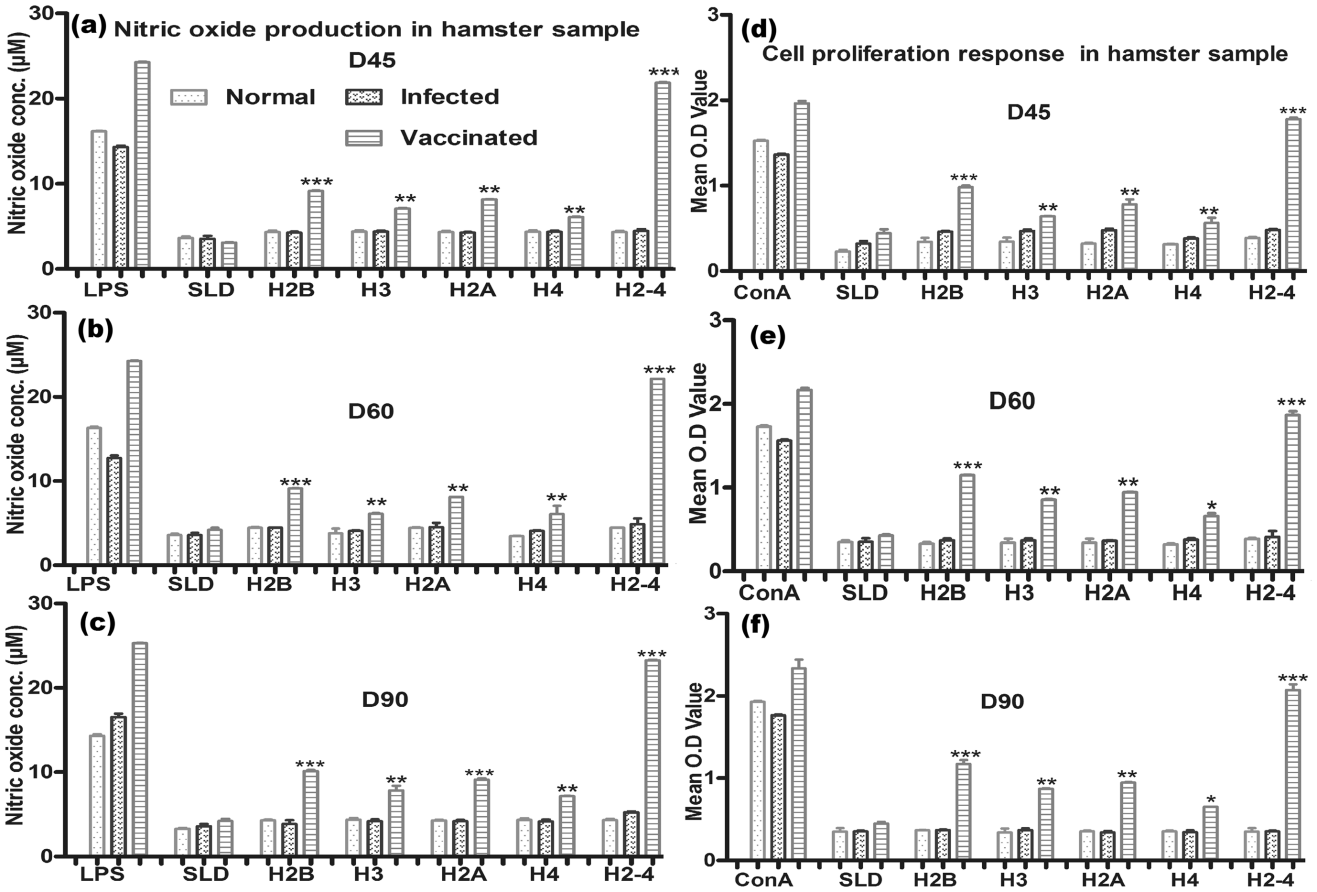
Cellular responses of rLdH2B, rLdH3, rLdH2A, rLdH4, and pooled (H2-4) of *L.donovani* in vaccinated hamsters. : (d,e,f) XTT response of mononuclear cells (lymph nodes) from normal, *L. donovani* infected and treated hamsters in response to Con A, SLD and rLdH2B, rLdH3, rLdH2A, rLdH4 and pooled (H2-4) at 10 µg/ml each. Proliferation was represented as mean OD. The data represent the means of triplicate wells ± the S.D. Nitric oxide production (µM) by (a, b, c) J774A.1 cell line. The J774 A.1 cells were primed with the supernatants of stimulated lymphocytes (3 days with mitogen and 5 days with antigens) of normal, infected and cured hamsters in response to rLdH2B, rLdH3, rLdH2A, rLdH4 and pooled (H2-4), SLD and LPS respectively at 10 µg/ml each. The estimation of NO production was done using Greiss reagent in supernatants collected from macrophage cultures 24 h after incubation and the absorbance of the reaction product was measured at 540 nm. Pooled (H2-4) shown significantly higher cellular response and nitric oxide production. Significance values indicate the difference between the SLD and rLdH2B, rLdH3, rLdH2A, rLdH4, and pooled (H2-4) stimulation (*, p<0.05; **, p<0.01; and ***, p<0.001).

NO production and lymphoproliferative response were also recorded to be significantly higher in hamsters immunized with pooled rLdH2-4 group wherein an optimum stimulation to the tune of 3.5 to 4.0 folds was observed in comparison to unvaccinated infected control group of hamsters and this was highly significant (P<0.001) (Fig 7). The cell proliferation and nitrite production in the hamsters response was moderate against all the individual proteins (1.2 to 1.5 fold). However, BCG vaccinated group exhibited parasite load and immune responses similar to infected control (Fig S1 and Fig S2).

### Immunization with rLdH2-4 generates Th1-type cytokine profile as determined by qRT-PCR

**Figure 8:**
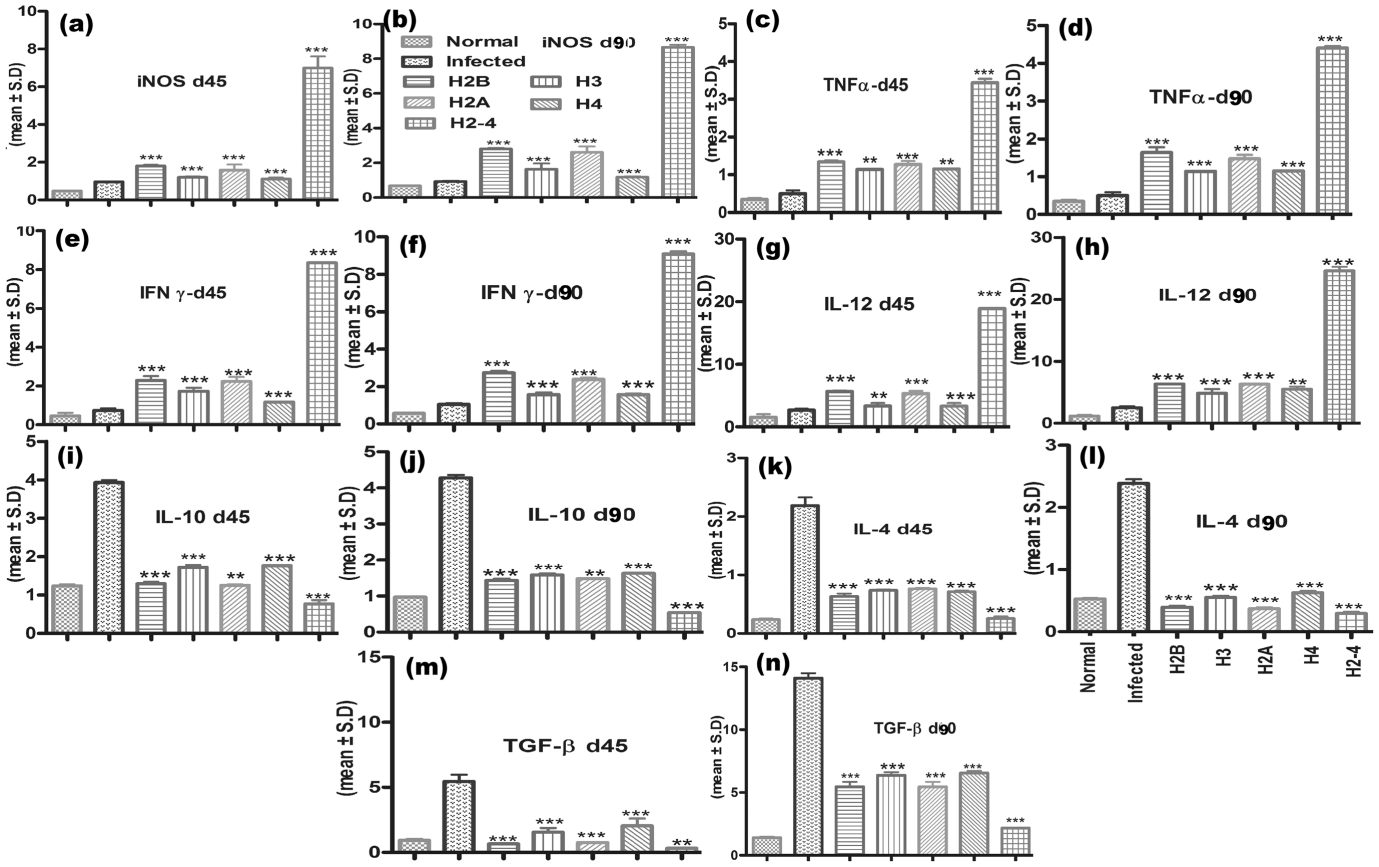
Splenic iNOS and cytokine mRNA relative fold expression profile analysis of normal and immunized hamsters on days 45 p.c. and 90 p.c by quantitative real-time RT-PCR. The expression of Th1 cytokines viz. TNF-α (c,d) as well as IFN-γ (e,f) was also observed to be ∼3.25 folds higher (p<0.001) in the hamsters of rLdH2-4 vaccinated group as compared to the other experimental as well as control groups. The expression of Th2 cytokines particularly IL-10 (i,j), IL-4(k,l) and TGF-β (m,n), known to be associated with progressive VL, were significantly down-regulated by ∼3.5 (p<0.01) to ∼4.5folds (p<0.001) in rLdH2-4 vaccinated hamsters and rLdH2B/rLdH3/rLdH2A/rLdH4 (∼1.5folds (p<0.01)). Significance values indicate the difference between the vaccinated group and infected group (**, p<0.01; and ***, p<0.001).

For further assessment of cellular immune response in hamsters immunized with rLdHistones either individually or in collective forms, the expression of Th1 and Th2 mRNA cytokines was measured by qRT-PCR. The iNOS transcript was found to be optimally and significantly upregulated by ∼5 folds (p<0.001) in hamsters immunized with rLdH2-4 as compared to that of *L.donovani* infected group followed by those immunized with rLdH2B/rLdH3/rLdH2A/rLdH4(∼2.0folds). The expression of Th1 cytokines viz. TNF-α as well as IFN-γ was also observed to be ∼3.25 folds higher (p<0.001) in the hamsters of rLdH2-4 vaccinated group as compared to the other experimental as well as control groups. Similar was the case with IL-12 expression which was also significantly (p<0.001) increased by ∼4.0 folds in rLdH2-4 immunized hamsters. On the other hand, the expression of Th2 cytokines particularly IL-10, IL-4 and TGF-β, known to be associated with progressive VL, were significantly down-regulated by ∼3.5 (p<0.01) to ∼4.5folds (p<0.001) in rLdH2-4 vaccinated hamsters and rLdH2B/rLdH3/rLdH2A/rLdH4 (∼1.5folds (p<0.01)) (Fig.8).

### Immunization with rLdH2-4 generates IgG2 type antileishmanial antibody response

**Figure 9:**
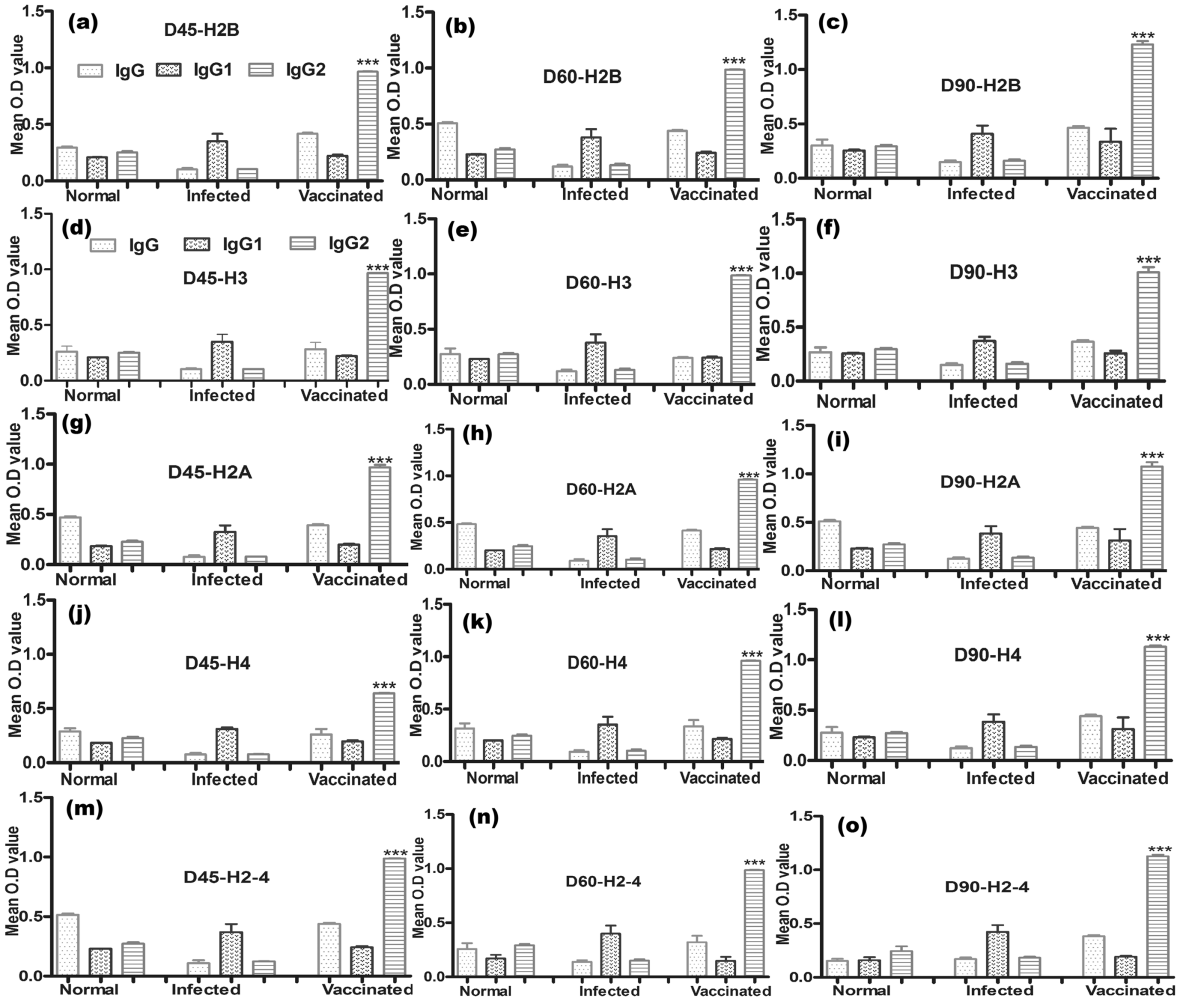
Antibody response of *Leishmania*-specific IgG and its isotypes IgG1 and IgG2 in rLdH2B, rLdH3, rLdH2A, rLdH4 and pooled (H2-4) of vaccinated hamsters in comparison to the unimmunized infected hamsters on days 45, 60, 90 p.c. Serum samples were collected from different groups of hamsters at designated time points and assayed for specific IgG, IgG1, and IgG2 levels by ELISA. Significance values indicate the difference between the vaccinated group and infected group (***, p<0.001).

The antileishmanial IgG and IgG1 were observed to be elevated by 2 to 3 folds in the infected control group in comparison to the group of hamsters immunized with rLdHistone and their pooled (rLdH2-4) wherein their levels were very low. On the other hand, there was significant elevation of IgG2 level (by 3 to 4 folds) in group of hamsters immunized with rLdHistone either individually or in collective forms (Fig.9). As a measure of cell mediated immunity (CMI), the elevation of IgG2 was indicative of the development of effective immune responses.

## Discussion

Histones are structural proteins and building units of nucleosomes which play an important role in DNA packaging, transcription and regulation of gene expression as well as the organization and function of DNA within the eukaryotic nucleus. Out of five types of histones that have been identified viz. H1 (or H5), H2A, H2B, H3 and H4, the latter four are associated with DNA to form nucleosomes. There are important differences between *Trypanosomatid* histones and their human counterparts [27]. First, *Trypanosomatid* histone genes are located on separate chromosomes, their transcripts are polyadenylated and their transcription is not always coordinated with DNA replication. Second, while the globular regions of the *Trypanosomatid* core histones are relatively conserved, the N-terminal domains of these chromosomal proteins are highly divergent. Taking this information into account, nucleosomal organization and function in *Trypanosomatids* may be quite different from that seen in humans and this knowledge could be advantageous in designing anti-infectives against these parasites specifically. Among the *trypanosomatids* the histones have been reported to be characterized in several sp of *Leishmania* viz *L.major* and *L.infantum* but no reports are there for *L.donovani* a causative agent of fatal anthroponotic VL. Herein, we have carried out molecular and immunological characterization of the histone proteins (H2B, H3, H2A, and H4) of *L. donovani.*

All the four core histone genes of *L.donovani* were cloned and sequenced using the primers designed on the basis of sequences of *L.major* and *L.infantum.* Of the four, H3 and H4 exhibited ∼90% homology with histones of *L.major* and *L.infantum* whereas the other two H2A and H2B were homologous to the tune of 82–85%. Similarly, a comparative sequence analysis with human histones have revealed that H2B protein of *L. donovani* have highly divergent region from human histone sequence but histone H3 and H4 were more homologus to the human histone.

There are reports regarding histone proteins of *L.major* and *L.infantum* as relevant immunogens for the host immune system during both *Leishmania* infection and disease [17], [18], [19], [28] as after parasite destruction or spontaneous lysis, exposure to these proteins elicits a strong host immune response. This prompted us to assess the immunogenicity of all the four recombinant histone proteins either individually and in pooled histone *in vitro*. Since, it has been observed that a T-cell response develops when *Leishmania* infected patients are cured and they eventually become immune to re-infection, [14], [29], the ability of all the four rLdHistone proteins to induce cellular immune response in *Leishmania* infected and cured hamsters was tested and then further validation of its immunogenicity in endemic non-immune donors (household contacts without any clinical symptoms) and in immune patients of VL that were cured either with amphotericin B or miltefosine, was done. The Th1 and Th2 subsets are very clear in rodent particularly in murine cells but not so in the human system and the infection pattern in murine VL also do not simulate the human profile, as it is self-limiting. On the other hand, systemic infection of the hamster with *L. donovani* is akin to human VL as it results in a relentlessly increasing visceral parasite burden, progressive cachexia, hepato-splenomegaly, pancytopenia, hypergammaglobulinemia, and, ultimately, death [26]. Hence, analysis of cellular immune response of the individual recombinants protein rLdH2B, rLdH3, rLdH2A and rLdH4 along with pooled (rLdH2-4) were carried out using lymphocytes of cured hamsters as well as human lymphocytes in order to correlate the observations made with both hamster and humans [14], [29]

During the leishmanial infections, macrophages become activated by IFN-γ derived from parasite-specific T cells, and destroy the intracellular parasites through the production of several mediators, prime among which is NO [30], [31]. In the absence of cytokine reagents against hamsters, we studied the effect of rLdhistone proteins on NO production. A significantly higher cellular responses viz. lymphoproliferative as well as NO release was observed against the pooled form of all the four recombinant histone proteins (rLdH2-4) in all the cured hamsters as compared to individual histone proteins. Similar observations were made in case of endemic control and cured patients of VL wherein strong T cell proliferation and IFN-γ production was induced by the pooled form of four histone proteins as compared to its other counterparts. Thus, individuals who control parasite burden successfully either following treatment in the case of patients or due to adequate immunity, as in endemic contacts, exhibit good T cell reactivity to the *Leishmania* histone proteins.

The presence of a positive immune response in all the eight endemic contacts indicates towards the high frequency of sub-clinical infection in an endemic area such as Bihar, India as has been reported in other endemic parts of the world [32], [33], [34]. It is well established that recovery from *Leishmania* infection, relies on induction of the Th1 response [35], [36] particularly the production of IFN-γ and IL-12 and improved expression of iNOS [30], [37]. Some of the recombinant antigens have previously been shown to induce lymphocyte proliferation and IFN-γ production in subjects cured of visceral and in patients with cutaneous or mucosal leishmaniasis [16], [32], [38].

SLD has been observed to stimulate PBMCs from *L. donovani*-infected individuals eliciting a mixed Thl- and Th2- type immune response, but in this study, we noticed that all the recombinant histone proteins of *L. donovani* particularly the pooled rLdH2-4 shifted this pattern towards an exclusive Thl (IFN-γ and IL-12) cytokine profile. These recombinant proteins stimulated PBMCs from cured and infected patients to secrete TNF-α and stimulated T cells from all the cured/infected patients to proliferate and produce IFN-γ associated with protective immunity. Cytokines appear to be essential mediators of immunity to *Leishmania* [12] wherein IFN-γ and TNF-α synergize to induce leishmanicidal activity in macrophages [39], [40], [41], [42]. In our study, TNF-α and IL-12 but not IL-10 were produced by cured/patient PBMCs stimulated with rLdHistones. This may be due to the relatively lower level of either TNF-α or IL-12 produced by patient PBMCs, as well as the ability of IFN-γ, produced in high amounts by patient PBMCs, to inhibit the production of IL-10 [43]. Further, it was observed that rLdHistone proteins down-regulated the levels of IL-10 in patient PBMCs. The use of patient PBMCs, rather than purified cell populations, may be more relevant to the *in vivo* situation since the dominant cytokine pattern is dependent on the interplay of modulatory cytokines. It appears that certain *Leishmania* antigens may be able to elicit a dominant Thl cytokine profile as well as inhibit the production of Th2 cytokines (IL-10) by mechanisms that are not fully understood, because IL-12 plays a central role in the initiation and maintenance of Thl responses in humans and mice [44], [45], [46], [47] and is a potent inducer of IFN-γ [31], which may inhibit the production of IL-10 [43]. IL-12 has been shown to play a pivotal immunoregulatory role in the development of cell-mediated immunity, including the generation of Thl responses and IFN-γ production in intracellular bacterial or parasitic infections [24]. rLdHistones stimulated consistently higher IL-12 production in cured and infected patient PBMCs than those observed with SLD respectively. This may suggest a role for IFN-γ in the SLD-induced IL-12 level observed in PBMCs of *Leishmania* patients, which produced higher IFN-γ than normal PBMCs after antigen stimulation.

Further, the prophylactic potential of rLdH2B, rLdH3, rLdH2A and rLdH4 and including pooled were assessed rLdH2-4 with the BCG adjuvant and observed that pooled rLdH2-4 with BCG adjuvant offered greater resistance to *Leishmania* challenge as an optimal reduction in parasite load (∼80%) in spleen, liver and bone marrow was observed on day 90 p.c which was significantly higher than all the other experimental as well control groups. This was followed by a prophylactic efficacy with individual rLdhistone protein (∼55%) in hamsters. Thus, the mixture of the four histones was clearly additive in the prevention of VL, which corroborate the similar findings [18], [19] in which histone proteins in cocktail form provide exclusive protection against *L.major* and/or *L.infantum* as compared to individual form. Cocktail of DNA vaccine coding for histone proteins (H2A, H2B, H3 and H4) was used to immunize BALB/c mice and led to an important Th1 cellular immune response and protection against *L. major* [18]. In another study, genetic immunization of BALB/c mice with the individual histones only resulted in a delay in lesion development, whereas the immunization with any one of the plasmids encoding a pair of histones provided stronger, though still partial protection against *L. major* infection as compared to the combination of the four histones. These results provide direct evidence that all the four nucleosomal histones of *Leishmania* are necessary to maintain complete protection against *L.major* reinfection [19]. In an another study on BALB/c mice, histones H2A, H2B, H3 and H4 of *L. infantum* elicited a specific Th1 immune response, which was associated with an antigen-specific production of interferon (IFN-γ) and a limited humoral response to histones (dominated by the antibodies of the IgG2a isotype) [18].

Nutshell of the study is that *L.donovani* histones results in a specific Th1-like response during infection and, it is providing exclusively protection with rLdh2-4 (cocktail). It's promising to be a good vaccine target.

## Supporting Information

Figure S1 **Clinical outcomes following** ***L. donovani*** **challenge in hamsters immunized with BCG alone.** On D 21 after the booster, the hamsters of infected, BCG alone groups were challenged intracardially with 10**^7^** metacyclic promastigotes of *L.donovani*. Parameters observed - Body weight (a), spleen weight (b), liver weight (c) and Parasite burden (no. of amastigotes per 100 cell nuclei) in the spleen (d), liver (e) and bone marrow (f) on D45, 60 and 90 p.c. Data represent mean values with standard errors (SE) at the designated time points in the experiments. The hamsters immunized with BCG alone and challenged with *L. donovani* exhibited similar parasite load in spleen, liver and bone marrow as infected.
(DOC)

Figure S2 **Antibody response of Leishmania-specific IgG and its isotypes IgG1 and IgG2 in BCG vaccinated hamsters in comparison to the unimmunized infected hamsters on days 45, 60, 90 p.c.** Serum samples were collected from different groups of hamsters at designated time points and assayed for specific IgG, IgG1, and IgG2 levels by ELISA. No Significance difference was observed between the BCG vaccinated groups and the infected group.
(DOC)
