# Correction: Nucleosomal Histone Proteins of *L*. *donovani*: A Combination of Recombinant H2A, H2B, H3 and H4 Proteins Were Highly Immunogenic and Offered Optimum Prophylactic Efficacy against *Leishmania* Challenge in Hamsters

**DOI:** 10.1371/journal.pone.0252177

**Published:** 2021-05-20

**Authors:** Rajendra K. Baharia, Rati Tandon, Amogh A. Sahasrabuddhe, Shyam Sundar, Anuradha Dube

After publication of this article [1], concerns were raised about Fig 1:

- Panels a, b, and d appear to present the same image.
- Panels k and l appear to present the same image.
- There appear to be discontinuities suggestive of image splicing in panels c (after M lane), f (after lanes labelled M, 3), i (after lanes labelled 1, 2), j, k, l (for j-l, discontinuities are before and after the lane labelled 1).
- The M Lane appears similar in panels j, k, l.

The authors noted that the whole panel duplications resulted from errors in figure preparation.

With this notice, the authors provide an updated version of Fig 1 and its legend. In the updated figure, panels b, c, d, k and l have been replaced with the correct images from the original experiments. Raw image data supporting the updated figure are in S1 File. Panels f-h have been replaced with photos of the original full-sized gels. Comparing the new versus original versions of panels f-h one can see where lanes were spliced out of panels f, g, h when the original published figure was prepared. The authors apologize for not having noted the splice lines on the original figure or in the accompanying legend. They did not provide clarifications in regard to the other image splicing concerns or the similarities in marker lanes between panels j and k, l.

As noted in the article [1], this study involved minor participants. Parents provided informed consent for these participants.

The individual-level data underlying other results reported in this article are no longer available.

**Fig 1:**
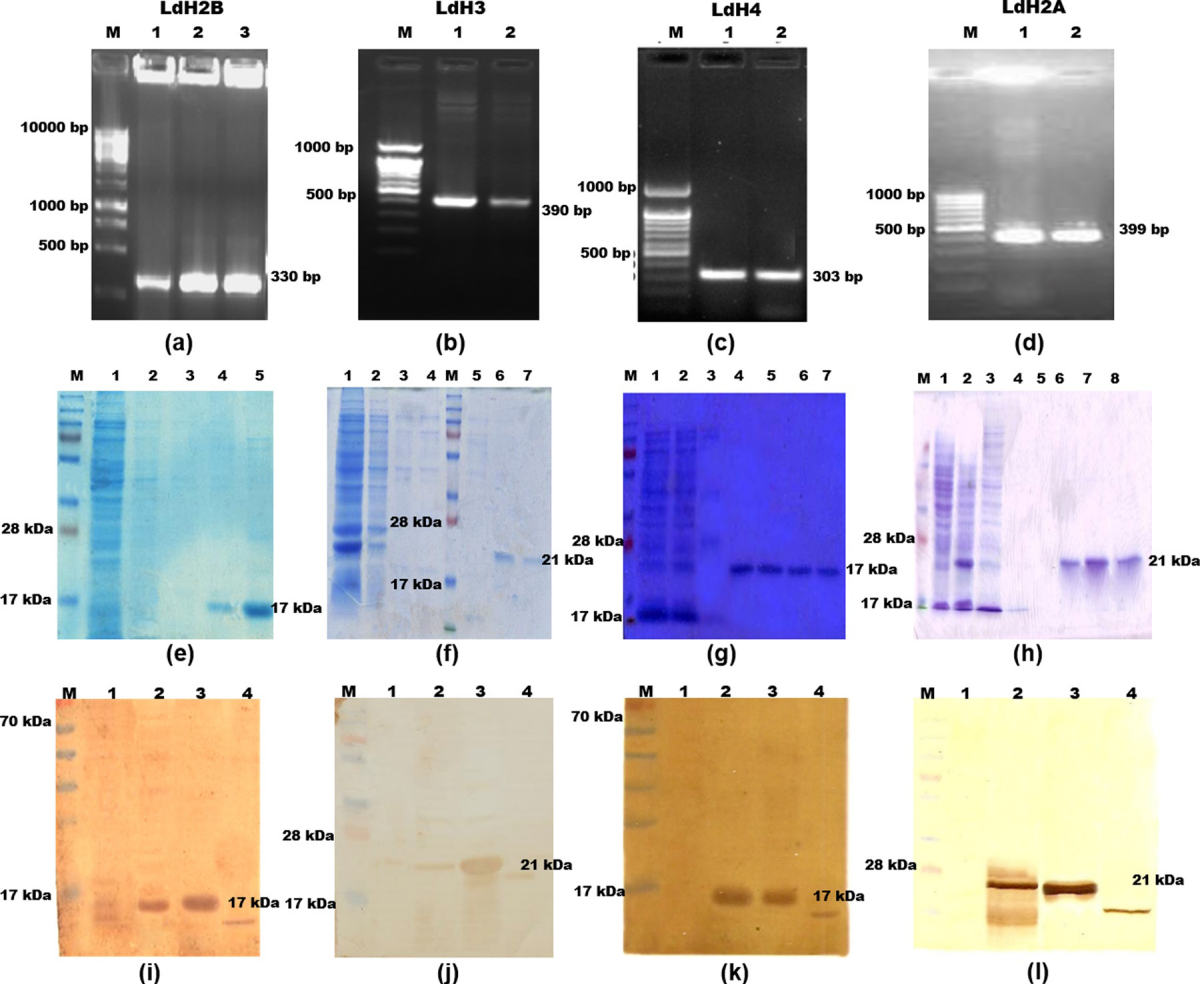
Updated Fig 1. (a,b,c & d)- PCR of of LdH2B, LdH3, LdH4 and LdH2A genes. (e, f, g, & h)- Expression and purification of LdH2B, LdH3, LdH4, LdH2A from *E*. *coli* rossetta cells, WCL of transformed *E*. *coli* separated on 12% acrylamide gel and stained with Coomassie blue, M: Molecular wt. markers; Lane 1: Flow through; lane 2&3: Washing fraction; Lane 4 & 5: eluted protein. (i, j, k & l)-Western blot analysis using anti-rLhistone antibody in uninduced WCL, induced WCL and *leishmania* WCL—M: Mol wt marker, Lane 1: uninduced WCL, Lane 2: induced WCL, Lane 3: Purified protein, Lane 4: whole cell lysate of *Leishmania*.

## Supporting information

S1 File Raw images provided in support of Fig 1. (PPT)
