# Supplementary figures and images for: Nucleosomal Histone Proteins of L. donovani: A Combination of Recombinant H2A, H2B, H3 and H4 Proteins Were Highly Immunogenic and Offered Optimum Prophylactic Efficacy against Leishmania Challenge in Hamsters
Source: PLoS One. 2014 Jun 13;9(6):e97911. doi: 10.1371/journal.pone.0097911 (PMC4057088; doi:10.1371/journal.pone.0097911)

**Figure S1**


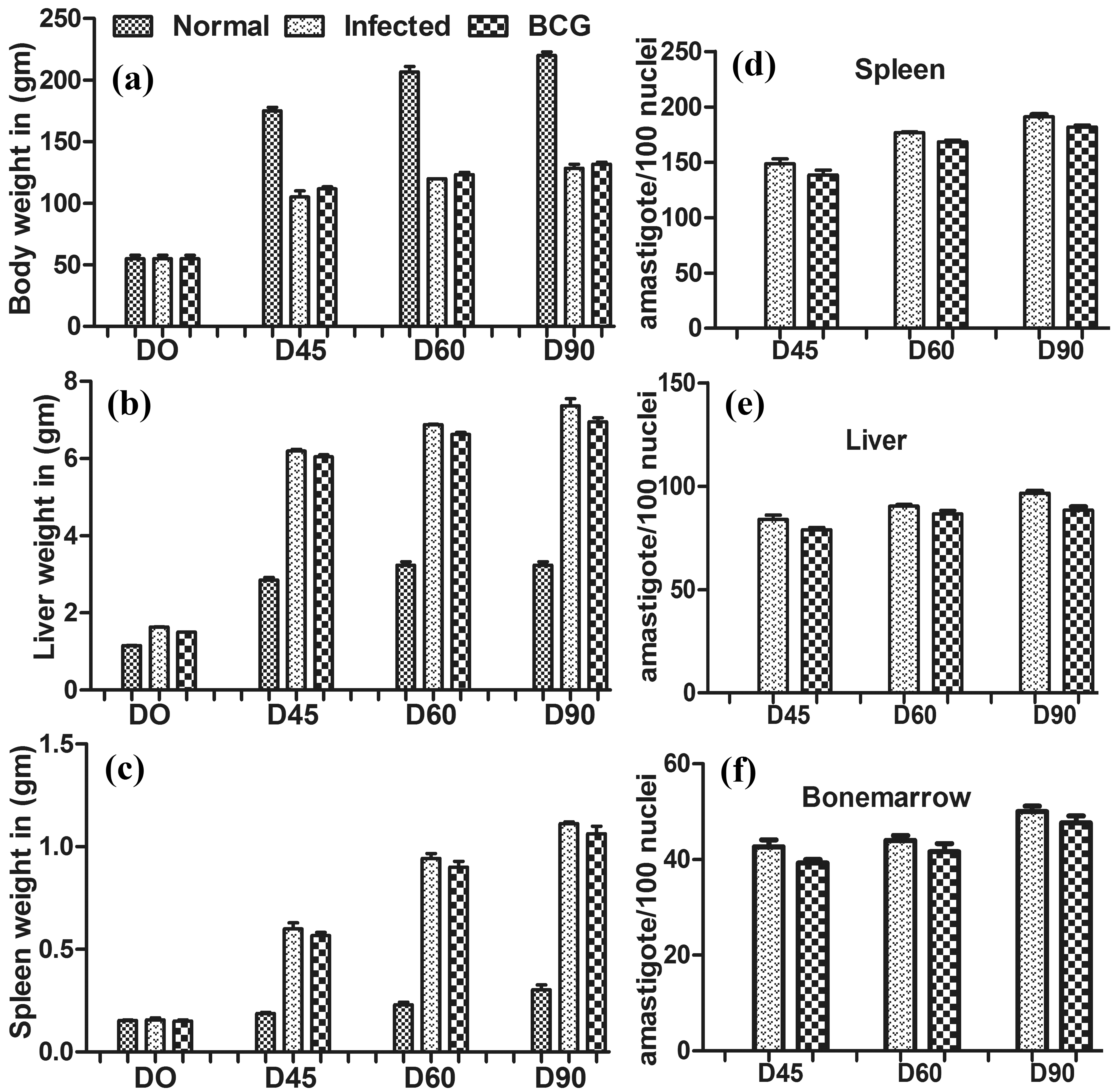

Supplement: Figure S1 — Clinical outcomes following L. donovani challenge in hamsters immunized with BCG alone. On D 21 after the booster, the hamsters of infected, BCG alone groups were challenged intracardially with 107 metacyclic promastigotes of L.donovani. Parameters observed - Body weight (a), spleen weight (b), liver weight (c) and Parasite burden (no. of amastigotes per 100 cell nuclei) in the spleen (d), liver (e) and bone marrow (f) on D45, 60 and 90 p.c. Data represent mean values with standard errors (SE) at the designated time points in the experiments. The hamsters immunized with BCG alone and challenged with L. donovani exhibited similar parasite load in spleen, liver and bone marrow as infected. (DOC) [file pone.0097911.s001.doc]

**Figure S2**


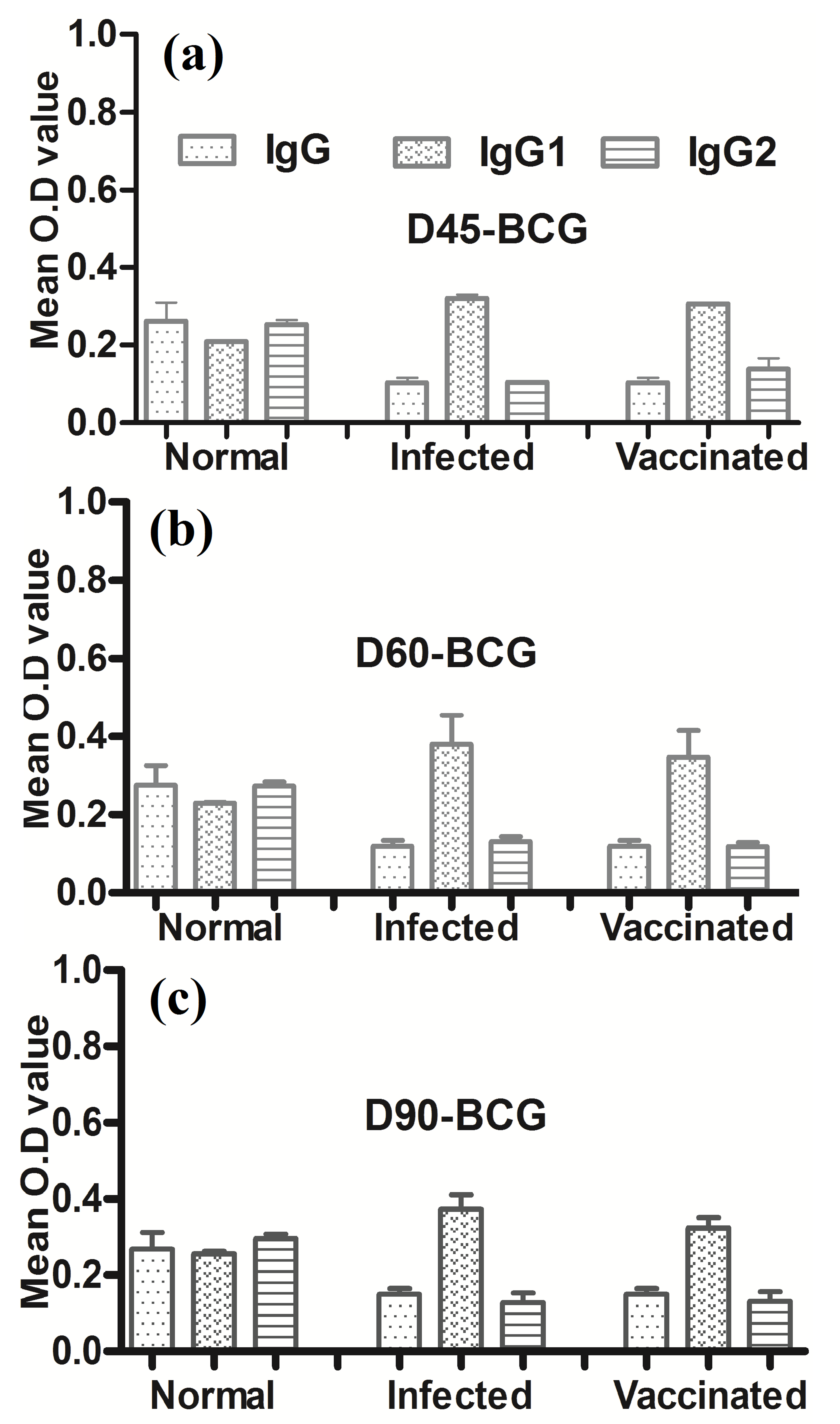

Supplement: Figure S2 — Antibody response of Leishmania-specific IgG and its isotypes IgG1 and IgG2 in BCG vaccinated hamsters in comparison to the unimmunized infected hamsters on days 45, 60, 90 p.c. Serum samples were collected from different groups of hamsters at designated time points and assayed for specific IgG, IgG1, and IgG2 levels by ELISA. No Significance difference was observed between the BCG vaccinated groups and the infected group. (DOC) [file pone.0097911.s002.doc]
