# Supplementary material for: Correction: Nucleosomal Histone Proteins of L. donovani: A Combination of Recombinant H2A, H2B, H3 and H4 Proteins Were Highly Immunogenic and Offered Optimum Prophylactic Efficacy against Leishmania Challenge in Hamsters
Source: PLoS One. 2021 May 20;16(5):e0252177. doi: 10.1371/journal.pone.0252177 (PMC8136653; doi:10.1371/journal.pone.0252177)
Supplement: S1 File — (PPT) [file pone.0252177.s001.ppt]

## Slide 1
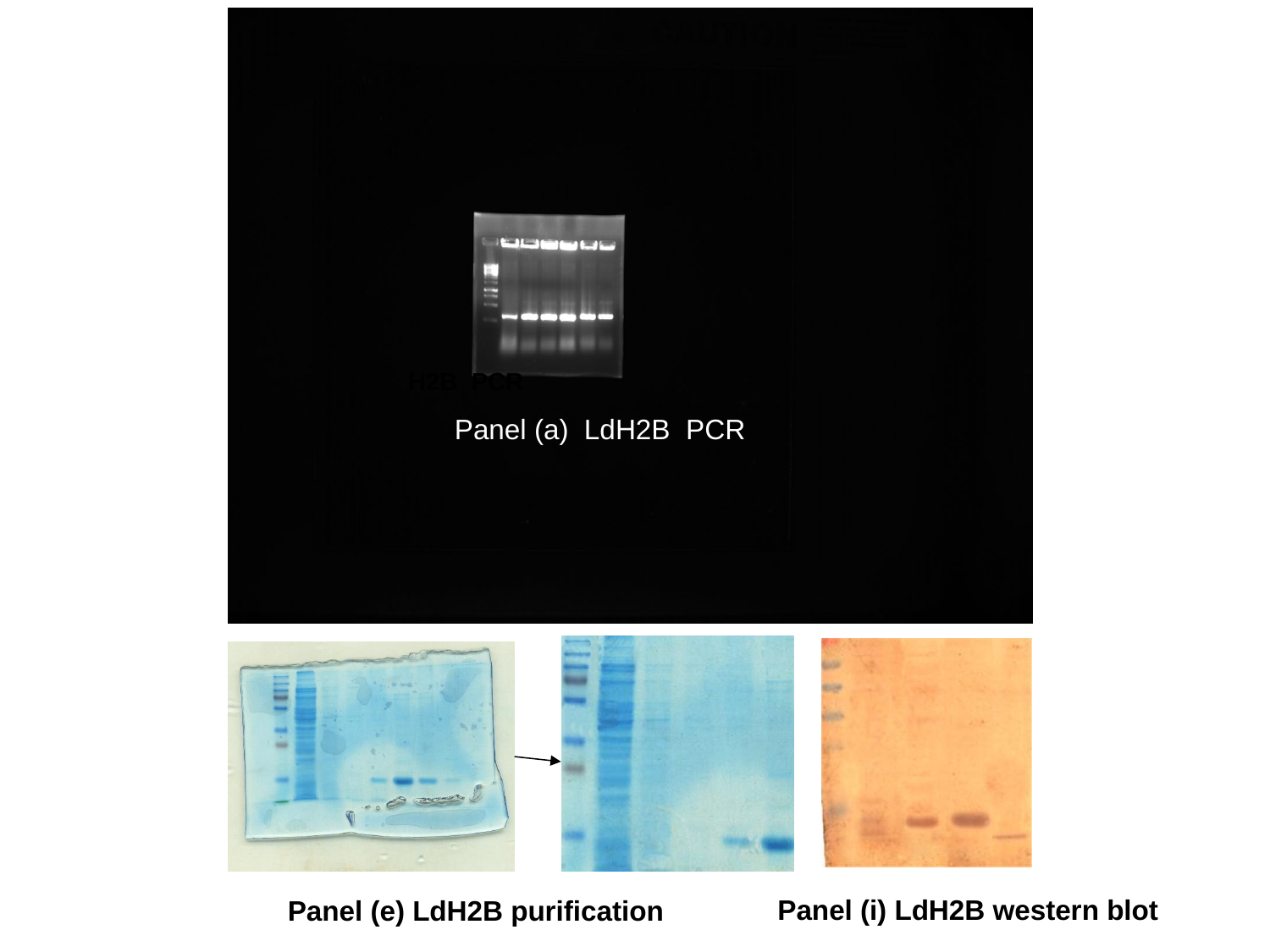

H2B PCR
Panel (a) LdH2B PCR
Panel (i) LdH2B western blot
Panel (e) LdH2B purification

## Slide 2
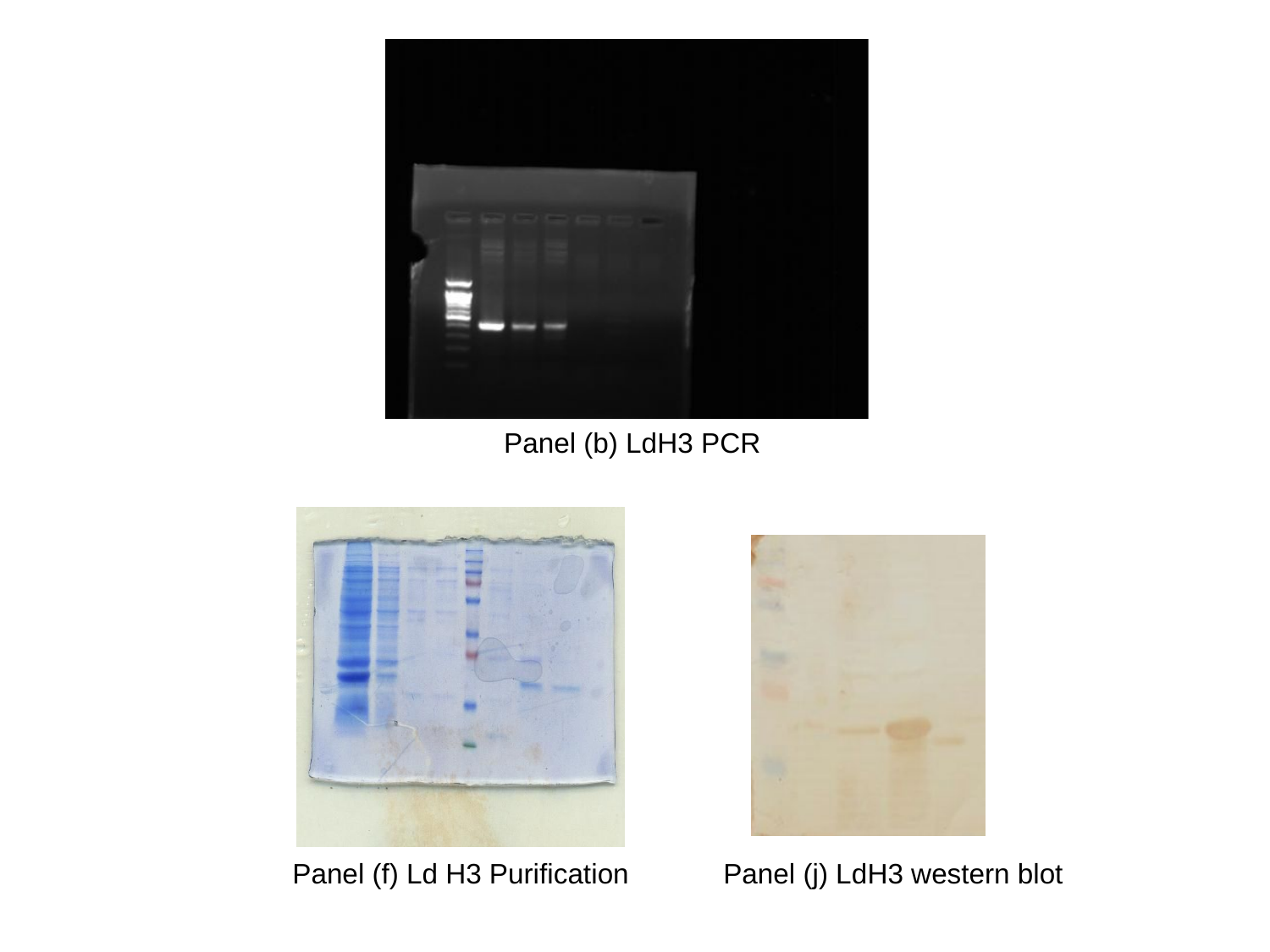

Panel (b) LdH3 PCR
# Panel (f) Ld H3 Purification
Panel (j) LdH3 western blot

## Slide 3
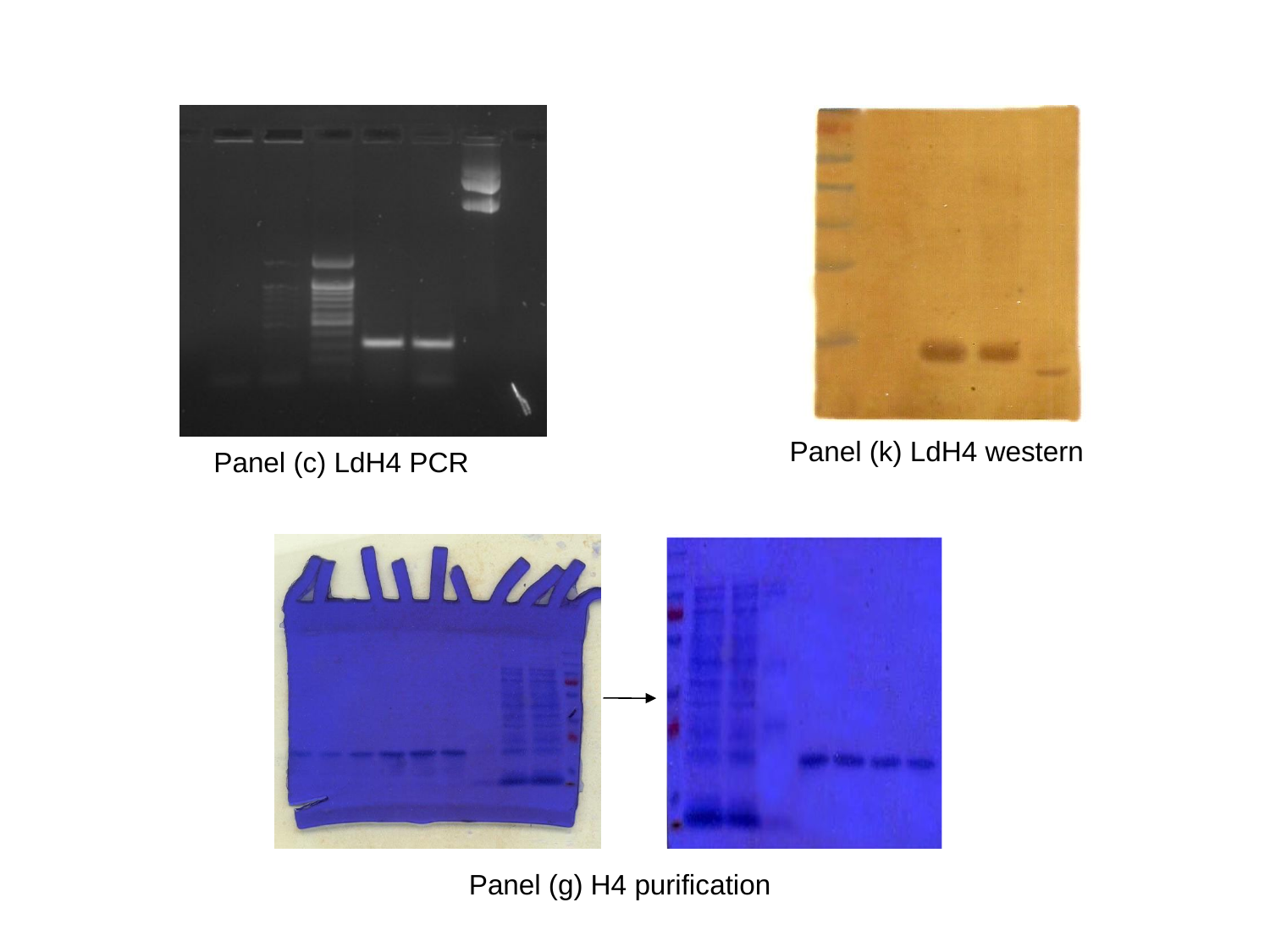

Panel (k) LdH4 western
Panel (c) LdH4 PCR
Panel (g) H4 purification

## Slide 4
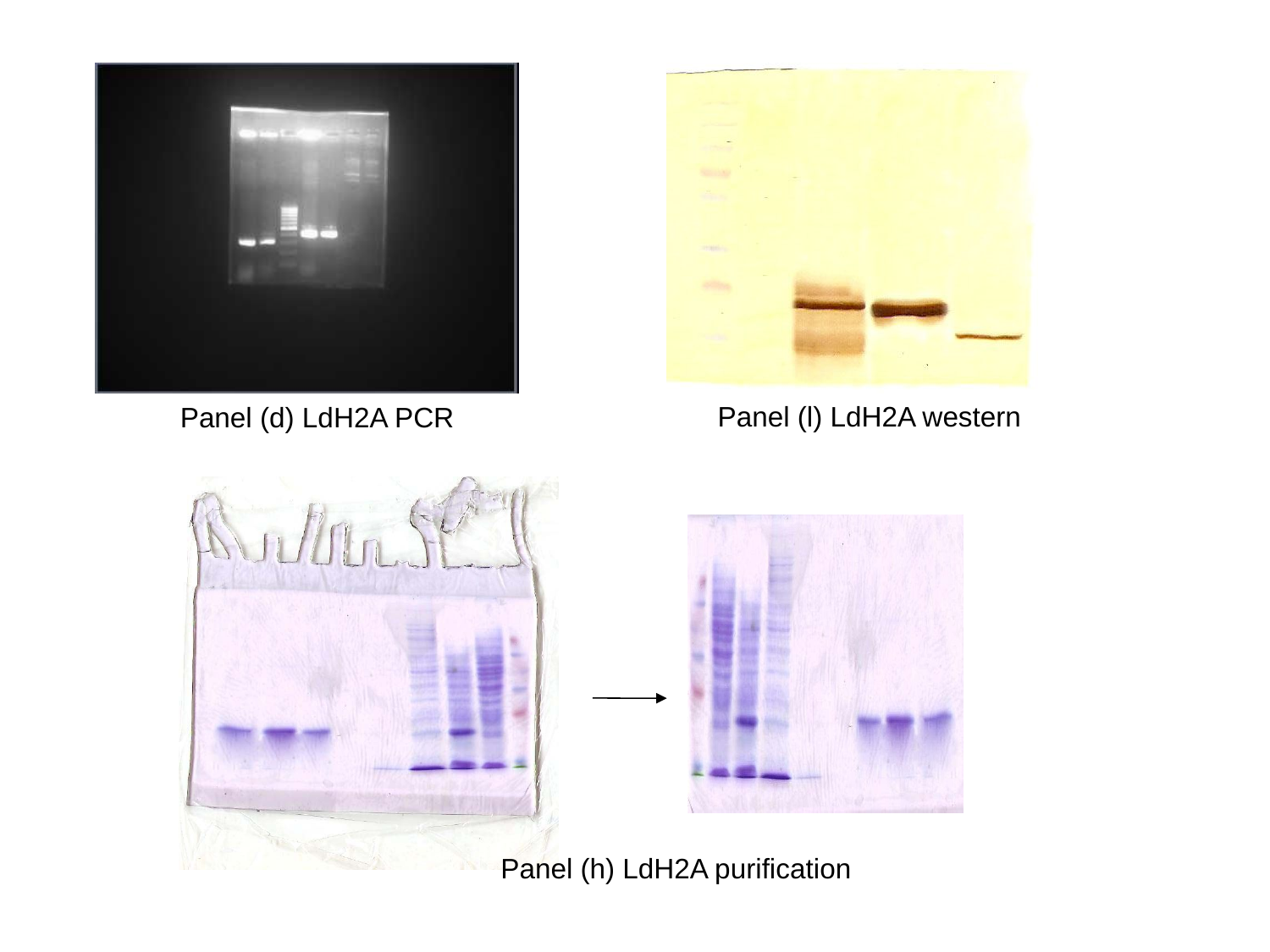

Panel (l) LdH2A western
Panel (d) LdH2A PCR
Panel (h) LdH2A purification
